# The emergence of urban scaling in Ancient Southwest Asia

**DOI:** 10.1371/journal.pone.0355479

**Published:** 2026-08-19

**Authors:** Francesca Chelazzi, Dan Lawrence

**Affiliations:** 1 Archaeology, School of Humanities, University of Glasgow, Glasgow, United Kingdom; 2 Department of Civilizations and Forms of Knowledge, University of Pisa, Pisa, Italy; 3 Department of Archaeology, Durham University, Durham, United Kingdom; University of Florida, UNITED STATES OF AMERICA

## Abstract

This study investigates the long-term emergence of settlement scaling in Ancient Southwest Asia by analysing house-size data from 1,852 buildings across 80 archaeological sites dating from the Late Epipalaeolithic to the Iron Age (c. 13,100–580 BCE). Using domestic architecture as a proxy for household productivity, we provide the first fully diachronic test of settlement scaling theory in the region where urbanisation first developed. Mixed-effects models reveal that scaling relationships were absent in the Neolithic, ambiguous in the Chalcolithic, and only fully emerged in the Bronze Age, where sub-linear exponents align with theoretical predictions for intensive socioeconomic outputs. These patterns persist into the Iron Age, indicating that strongly mixing interaction networks—central to settlement scaling theory—became structurally embedded only with the rise of urban complexity. Across periods, baseline house size more than doubles, suggesting long-term technological or organisational improvements independent of agglomeration effects. We also examine how scaling relates to economic inequality using Gini coefficients of residential size distributions. Inequality correlates with settlement size only in politically complex systems and primarily at major centres, indicating that institutional structures—not agglomeration-driven productivity—govern the allocation of returns. Residuals from scaling models do not predict inequality, demonstrating that productivity gains and their distribution operate through independent channels. Finally, we derive period-specific estimates of population density from the intercepts of area-population scaling relationships. These results document a marked decline in residential density from the Chalcolithic to the Bronze Age, followed by partial recovery in the Iron Age, aligning with independent reconstructions from sites such as Tell Brak. Overall, the study shows that scaling behaviours emerged only with sustained urban integration, and that inequality in early cities was shaped more by political institutions than by the geometric constraints of urban interaction.

## Introduction

Understanding how cities function, how their properties change with scale, how they concentrate and distribute resources, and how they create both opportunity and inequality, is a major challenge in social sciences. With urban populations projected to approach 70% of the global total by mid-century [1], the challenge is especially urgent today. However, the processes that make cities function as they do are not exclusively modern: cities have existed for over six millennia, and the social dynamics that structure urban life—the concentration of interaction in space, the division of labour, the tension between agglomeration benefits and spatial costs—existed already in the earliest urban centres of Mesopotamia, the Indus Valley, and Mesoamerica [2]. Archaeology, as the only discipline that can observe these processes over the longue durée, holds a unique position, able not merely to apply urban theory to the past, but to contribute to its development by revealing which properties are structural to urban life and which are contingent on specific energy regimes and institutional conditions [3,4].

Settlement Scaling Theory (SST) offers an explicit quantitative framework to accomplish this task. Developed over the past two decades [4–9], SST proposes that the measurable properties of human settlements vary systematically with population size according to power-law relationships. The key parameter is the scaling exponent β, whose value depends on the type of quantity measured. For aggregate infrastructure, such as roads and built areas, SST predicts economies of scale where larger cities require relatively less infrastructure per capita. For aggregate socioeconomic outputs, such as total wealth or production, it predicts increasing returns to scale driven by social interaction. For intensive per capita quantities such as household productivity, it predicts that individual returns increase with settlement size but at a diminishing rate. These regularities are not assumed but derived from first principles. SST models settlements as social reactors in which per capita productivity is proportional to the average rate of social interaction, which in turn depends on population density and the spatial organisation of the settlement.

What makes SST distinctive is not the identification of power-law regularities per se—such patterns have been noted since at least Nordbeck [10]—but the derivation of the numerical values of β described above from first principles of social interaction in space [6]. SST does not predict that power-law relationships will be found wherever one looks, but rather specifies the conditions under which they should arise—strongly mixing social networks functioning inside bounded spatial containers—and predicts the parameter values when those conditions are met. The theory is therefore generative precisely when it encounters the unexpected, as deviations from predictions are not failures but diagnostic signals. This is particularly powerful for archaeological examples where spatial interaction cannot be observed and must be inferred from material remains. For example, Smith et al. [11] found that dispersed Maya settlements show de-densification instead of the expected densification, indicating that these settlements did not function as the kind of mixing networks SST presupposes, while the public ritual centres where people periodically gathered did show the expected pattern. Smith et al. [11] use this result to argue that Classic Maya populations did not mix socially on a daily basis across their settlements, but instead congregated periodically in civic-ceremonial centres, a finding that redefines what counts as the relevant ‘settlement’ for scaling analysis.

The SST predictions have been confirmed across dozens of contemporary urban systems worldwide [5,8,12] and subsequently tested in a growing range of archaeological contexts: pre-Hispanic Mesoamerica [7,9], the Central Andes [13], medieval Europe [14], the Greco-Roman world [15,16], and middle-range village societies in North America [17]. A crucial advantage of these archaeological applications is that the physical boundaries of ancient settlements correspond more directly to functional boundaries than is typically the case for contemporary cities, where defining functional urban areas remains a persistent challenge [18,19]. In preindustrial societies, where movement was restricted to walking, animal or water-borne modes and most interactions were confined to the settlement, the physical and functional settlement were essentially one and the same [3], giving the archaeological record a distinctive analytical advantage for the study of scaling phenomena.

Two important dimensions of SST remain underexplored, however, and both are uniquely accessible through archaeology. The first is the temporal emergence of scaling: when, in the deep history of a given region, do the social and spatial conditions for scaling first arise? The second concerns the relationship between scaling and inequality: SST predicts that larger settlements generate disproportionately greater aggregate wealth, but whether this translates into greater inequality within settlements—and under what institutional conditions—is a question the theory does not currently address, as it operates at the level of aggregate settlement outputs rather than their internal distribution [18]. A related assumption in economics holds that greater aggregate wealth should generate greater inequality, simply because there is more wealth available to be distributed unequally, a constraint formalised as the inequality possibility frontier (IPF) [20]. The IPF assumes that the maximum achievable inequality in a society is bounded by its average productivity, since a population cannot be both very poor and very unequal without collapsing. However, recent archaeological work has shown that this frontier is mediated by institutional arrangements including political hierarchy, modes of governance, and the functional position of settlements within regional systems [21–23].

Southwest Asia provides an unparalleled laboratory for both questions. As one of the world’s primary centres of early urbanisation, it encompasses the full trajectory from mobile hunter-gatherer camps through the earliest sedentary villages, the first urban agglomerations during the fourth millennium BCE, and the imperial cities of the Bronze and Iron Ages, a span of over twelve millennia during which virtually every innovation SST identifies as relevant to the emergence of scaling appeared for the first time [24–29]. The region witnessed multiple pathways to urbanisation [30], with diverse regional trajectories including integrated systems with successful administrative centralisation, alongside areas where urban centres remained weakly connected to their rural hinterlands, with limited economic and political integration beyond the city itself [31–34]. The nature of the available evidence in Southwest Asia has meant that scholars have struggled to identify variables suitable for scaling against settlement size. In other regions, such as the Roman world or Mesoamerica, data have been collected at sufficient scales to enable analyses of variables like monument construction rates or plaza size [7,16], evidence types that are clearly delineated in those urban traditions but largely absent from the more organically structured townscapes of Southwest Asia. In Southwest Asia, by contrast, such systematic datasets are largely absent. Where SST has been applied to the region, it has focused on transport infrastructure [35], street network centrality [36], or the pre- and post-imperial periods alone [37], rather than examining the emergence of scaling behaviours over deep time. The tell-based archaeology of the region—with its deeply stratified and multi-period mounds—partly explains this gap, as the exposure of large single-phase areas is exceptionally difficult. Excavation has also overwhelmingly prioritised monumental over residential architecture [38,39].

To address the absence of a fully diachronic test of SST in Southwest Asia, this paper uses residential architecture as a common empirical measure through which to examine the long-term relationships between population, productivity, inequality, and urban form. Drawing on house-size data from settlements spanning the entire trajectory of Southwest Asian urbanisation, we first evaluate whether and when settlement scaling relationships emerge, and whether their parameters conform to theoretical expectations. We then use the same dataset to investigate patterns of household inequality and their relationship to settlement size, productivity, institutional complexity, and settlement hierarchy. Finally, we show how SST provides a framework for deriving estimates of population density from archaeological data, allowing comparison across periods and regions. The central methodological premise of the paper is that residence size captures a household’s long-term productive capacity, making it possible to integrate scaling, inequality, and demographic analyses within a single analytical framework.

The ubiquity of housing in excavations from all periods enables for the first time a fully diachronic analysis of scaling relationships across the entire temporal spectrum of Southwest Asian urbanisation. It is important to clarify the position of house size within the analytical framework of this study, as the same variable serves different purposes in the scaling and inequality analyses. Within SST, residence size is treated as a proxy for the productivity of the associated household unit, while mean residence size across a settlement or region is interpreted as mean productivity. The implication of this assumption is that mean house size fits into SST as a quantity that should scale with population at approximately 1/6 [6,7]. Ortman et al. [7] make this explicit by treating “the surface area of a domestic residence as a proxy for the productivity of the associated household unit” while Ortman et al. [13] measure household productivity in terms of roofed space. This is categorically distinct from ‘infrastructure’ in the SST framework (e.g., roads, access networks, and public spaces) for which the predicted exponent is 5/6 [6,18].

Our analysis thus employs the same variable for two complementary purposes: scaling analysis uses mean residence size as a measure of intensive productivity, while inequality analysis uses the within-site distribution of residence sizes as a measure of economic disparities between households. Although these two uses might appear to invoke different economic concepts—productivity on the one hand, and wealth inequality on the other—both converge on a common underlying quantity. The concept of permanent income, developed by Friedman [40] to explain why household consumption reflects long-term expectations rather than current income, provides a unifying framework. Constructing a house requires the sustained mobilisation of labour, materials, and social relationships over time; it is therefore an investment that reflects a household’s expected long-term productive capacity rather than its current short-term income or longer term stock of accumulated wealth. From this perspective, mean house size captures the aggregate medium-term productive capacity of a community—precisely the quantity that SST predicts should scale with settlement size as an intensive socioeconomic rate—while the distribution of residence sizes captures how that capacity is allocated among households, providing a measure of economic disparity. The two analytical frameworks thus share a common empirical foundation, and the apparent tension between their uses of house size dissolves when both are understood as different expressions of the same underlying variable: the capacity of households to generate and sustain resources over time [41].

Three substantive questions guide our analysis. First, *when do scaling relationships emerge in the archaeological record of Southwest Asia, and what does this timing reveal about the preconditions SST identifies as necessary for agglomeration effects?* The archaeological implications of this are that settlement systems that do not exhibit scaling may not yet have functioned as integrated interaction networks, analogously to the Maya case [11]. Second, *how do the observed scaling exponents compare with theoretical predictions and other archaeological contexts, and what do the deviations reveal about the regional specificity of tell-based urban forms?* If urban geometry determines scaling parameters [42], then morphological variation within Southwest Asia—from the compact tells of southern Mesopotamia to the more dispersed settlement patterns of the Levant—should produce systematically different exponents across sub-regions, a prediction we test through geographical comparison. Third, we ask *to what extent the relationships between settlement size, productivity and inequality are mediated by institutional complexity and functional position within settlement systems?* SST does not generate explicit predictions about the relationship between scaling and within-settlement inequality, treating this as a question that moves beyond mean-field approximations to incorporate contextual variables including culture, institutions, and place [18]. Our data allow us to investigate this relationship directly, examining both whether institutional complexity and hierarchical position mediate the inequality-size relationship, and whether settlements that are more productive than expected given their size—and therefore potentially have greater surplus available for unequal distribution—are also more internally unequal. Understanding whether agglomeration-driven productivity and inequality are structurally coupled or institutionally mediated has direct implications beyond the ancient world. If the two operate through independent channels, the apparent trade-off between agglomeration-driven growth and equitable outcomes dissolves: growth does not mechanically generate inequality, and institutional design becomes the critical lever for contemporary urban policy.

Beyond these three substantive questions, our dataset also allows us to address a long-standing methodological question in Mesopotamian archaeology: *how many people lived in these settlements per unit area?* Population density estimates for Ancient Southwest Asia have ranged from 40 to over 1200 persons per hectare depending on assumptions about residential proportion, contemporaneity, and site type [29,43–45], with no formal way of deriving the relationship between settlement area and population density from the data themselves. SST offers a framework for doing precisely this. The intercepts of the density and output scaling relationships predicted by SST can be read directly as estimates of households per hectare and total roofed residential space at a standardised settlement size, allowing us to test whether population density varies systematically across periods and to compare our estimates with both archaeological and textual evidence.

## Materials and methods

The analytical framework employed here draws on the formal mathematical structure of SST, which models settlements as social reactors in which aggregate output varies with population size according to power-law relationships of the form


$$\begin{array}{c} Y\left(N_{i},t\right)=Y_{\text{0}}(t)N_{i}^{\beta (t)}e^{\xi_{i}} \end{array}$$ (1)

where Y represents any measurable settlement attribute, Nᵢ is the population of settlement i, Y₀(t) is a time-dependent prefactor that reflects baseline productivity and interaction costs, β is the scaling exponent, and ξᵢ captures settlement-specific deviations from the expected relationship [46]. The β value is the essential metric for assessing scaling behaviour, but its interpretation depends on the type of quantity measured. For aggregate infrastructure (e.g., roads, networks, built areas), SST predicts β ≈ 5/6 to reflect economies of scale where larger cities require relatively less infrastructure per capita. For aggregate socioeconomic outputs (e.g., total wealth, total production, but also negative outputs such as crime rates), β ≈ 7/6, reflecting increasing returns to scale driven by social interaction. For intensive (per capita) quantities such as average household productivity, β ≈ 1/6, indicating that individual returns increase with settlement size but at a diminishing rate. Linear scaling (β = 1) indicates quantities that scale proportionally with population [5,6].

The theoretical foundations for these regularities derive from a model of settlements as spatial equilibria. According to SST, cities function as ‘social reactors’ where the aggregate output of a settlement can be expressed as


$$\begin{array}{c} Y_{i}\left(N_{i},t\right)=\frac{G(t){\left[N_{i}(t)\right]}^{\text{2}}}{A_{ni}(t)}\cdot e^{\xi_{i}(t)} \end{array}$$ (2)

where [Nᵢ(t)]^2^ represents the total number of possible interactions within the population, Aₙᵢ(t) is the area over which interactions take place, and G(t) represents the social and technological factors that convert interactions into output [47]. Given the frictional effects of distance, this formulation explicitly states that per capita productivity is proportional to the average interaction rate. This relationship simplifies to the standard scaling form where β emerges from the geometry of how social networks fit within built space according to derivations that express interaction area as a function of population under the constraint that human effort is bounded [6,9]. By providing micro-foundations for aggregate scaling phenomena in terms of individual agent behaviour and their economic and non-economic interactions, these assumptions connect SST to long-standing research traditions in anthropology, sociology and economic geography [8].

**Fig 1:**
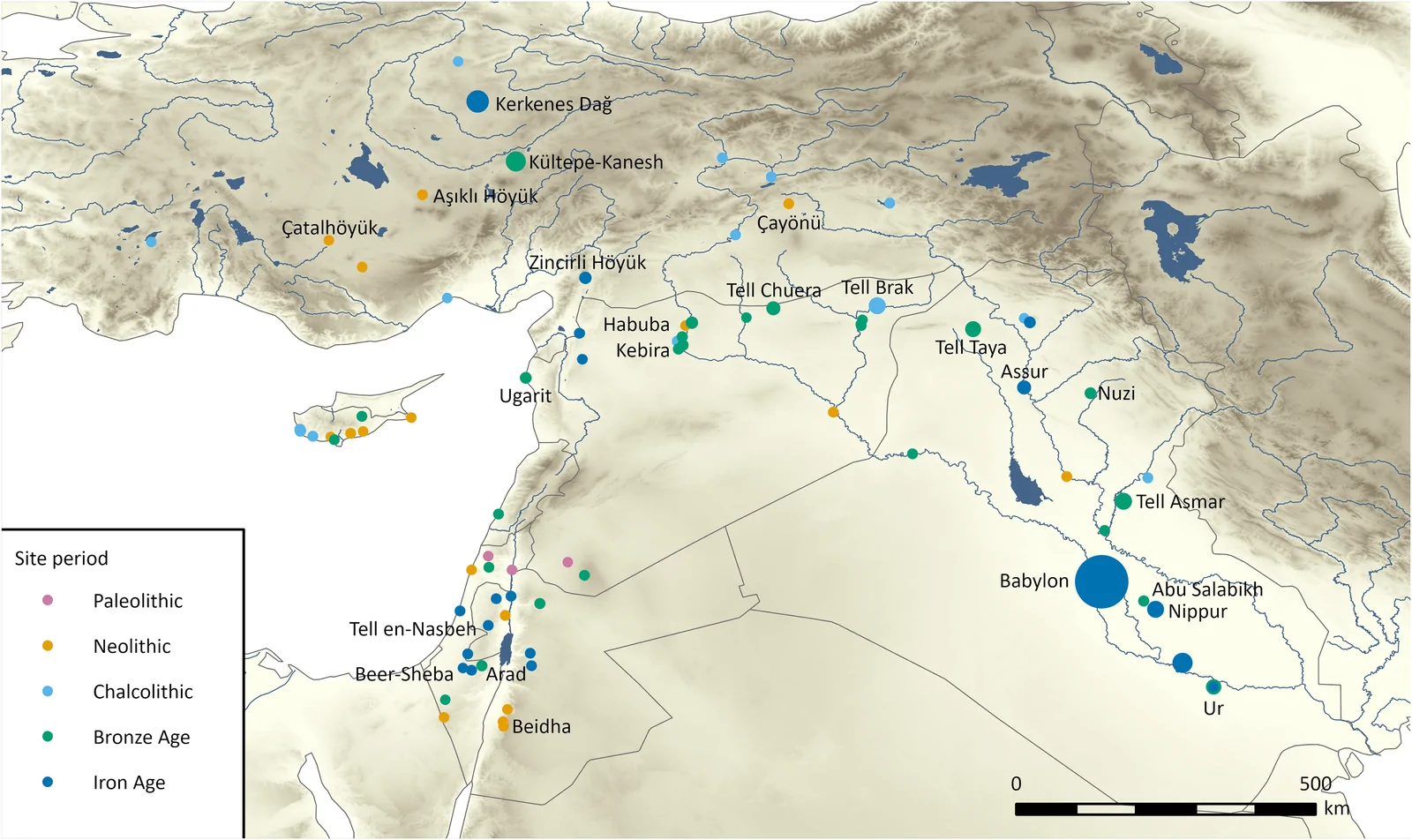
Map displaying the sites analysed in this study. Colours represent the temporal distribution of site phases, whilst symbol size reflects settlement area. Elevation data from GTOPO30, courtesy of the U.S. Geological Survey.

Our dataset comprises 1,852 buildings from 80 archaeological sites across Southwest Asia (southern Turkey, Syria, Lebanon, Jordan, Israel-Palestine, Cyprus, Iraq; Fig 1), spanning 107 site phases from the Late Epipalaeolithic (Hayonim Cave, 13,100−11,500 BCE) to the Iron Age (Kerkenes Dağ, 580−550 BCE). The Epipalaeolithic represents the earliest period for which residential structures are available in the region. The Iron Age marks the effective upper limit of the dataset. Beyond the Iron Age, tell-based settlement systems were progressively abandoned as regional trajectories diverged—the Levant becoming integrated into the Graeco-Roman urban tradition, Mesopotamia developing under Seleucid, Parthian and Sasanian influence—rendering a unified diachronic analysis of the kind attempted here no longer feasible. This provides comprehensive coverage of the region’s major cultural and technological transitions across one of the world’s primary centres of early urbanisation.

Data derive from the Global Dynamics of Inequality (GINI) Project database [48], to which the bulk of the Southwest Asian residence size data were contributed by the authors. The database was compiled through site monographs, excavation reports, and thematic studies representing decades of archaeological investigation, expanding on the 55 site phases analysed by Basri and Lawrence [38]. From the original GINI Southwest Asian dataset, we excluded Egyptian and Iranian data, as well as the site of Dura Europos (post-Iron Age) due to limited representativeness. We supplemented the dataset with Neo-Babylonian phase houses from Uruk and Nippur [37] to ensure adequate representation of major southern Mesopotamian urban centres, as our Iron Age sample otherwise consists predominantly of smaller Levantine rural sites. Following the GINI Project inclusion criteria, only site phases with a minimum of five measured residential buildings are included [48]. The GINI database also includes several contextual variables enabling multi-scalar analysis, such as polity type classifications following Johnson and Earle [49], settlement hierarchy position, and agricultural regime (i.e., land-limited vs labour limited) [50]. This study is based entirely on previously published archaeological data; no human remains or physical specimens were analysed, and no new fieldwork, excavation, or sampling was undertaken. No permits were required for the described study, which complied with all relevant regulations. Additional information regarding the ethical, cultural, and scientific considerations specific to inclusivity in global research is included in the Supporting Information (S2 File).

House size data from Southwest Asia are unevenly distributed in space and time, reflecting excavation biases and research traditions. Large exposure of single architectural phases are generally rare, as many sites consist of tells, settlement mounds where older phases are buried beneath later strata and only occasionally exposed in small sondages. Furthermore, archaeological exploration has been limited by modern occupation, and regional research trajectories have foregrounded particular periods and site types [38].

A fundamental characteristic of this dataset is that the chronological periods used in the analysis are composite categories, grouping sites from different sub-regions that were not necessarily contemporaneously occupied and did not form parts of a single interacting settlement system. This design is necessitated by the uneven distribution of domestic exposures across Southwest Asia. SST has not been used to compare settlements in this way before, and the approach constrains the types of questions the data can address. We are comparing within-period scaling estimates which represent averages across diverse settlement systems rather than parameters of any single contemporaneous network, and as such our dataset cannot be used to track the economic trajectory of an individual regional system through time, but rather to document broad temporal patterns in scaling behaviour across the region’s urbanisation sequence. The implications of this for the interpretation of scaling parameters are discussed in the Discussion.

The dataset focuses on residential architecture, excluding obvious public buildings and religious structures. However, some ambiguous cases of elite residences and palatial complexes are retained, recognising that the boundary between domestic and palatial architecture can be fluid, particularly in contexts of emerging social complexity. For scaling analysis, we used total house area measurements, which represent ground floor areas unless explicitly noted otherwise; multi-storey structures are calculated using ground floor dimensions to maintain consistency across sites with varying preservation conditions. House size distributions inherently include variability reflecting different household compositions, economic statuses, social roles, building materials and technologies within ancient communities. It must be remembered, however, that house data may not accurately represent all socioeconomic classes, as residential houses of lower classes were likely built with smaller budgets, reducing preservation likelihood. Furthermore, we likely miss ‘the missing zeros’ [51] such as slaves, property-less people, and nomadic communities, whose existence is amply supported by textual and archaeological evidence from Bronze and Iron Age Southwest Asia [52–55].

Scaling analysis requires two variables: a measure of settlement output and a measure of settlement population. Residence size—operationalised as total ground floor area as described above—serves as the former. For the latter, population estimates are unavailable for most sites in our dataset. We therefore use settlement area in hectares as a proxy for population size, following the approach adopted in previous scaling studies of Southwest Asian settlements [35,37] and consistent with the well-documented relationship between the two (*A* ∝ *N*^2/3) [3,9]. This substitution means that the expected exponent for house size against settlement area is approximately 1/4 rather than 1/6. Phase-specific site dimensions are used where settlements changed size during occupation, and for both variables we relied on measurements provided by original excavators and surveyors.

Our scaling analysis investigates the relationship between house size and settlement size through the following log-linear model:


$$\begin{array}{c} (HouseArea)=\alpha +\beta \times (SiteArea)+\varepsilon \end{array}$$ (3)

We use base-10 logarithms throughout because both house sizes and settlement areas are heavily right-skewed in their original scale, with log-transformation normalising this asymmetry. Settlement areas additionally span nearly four orders of magnitude (0.2–900 ha), making the base-10 scale more readily interpretable in graphical displays. The choice of logarithmic base does not affect the estimated scaling exponent β.

Fitting this model requires accounting for the hierarchical structure of the data, with multiple individual buildings measured within each site phase. Because houses within the same site phase generally share local conditions (e.g., construction traditions, available materials, social organisation, excavation exposure) they cannot be treated as independent observations. A standard ordinary least squares (OLS) regression would ignore this non-independence, underestimating standard errors and inflating the apparent significance of scaling relationships. We therefore employ linear mixed-effects models as our primary analytical tool (lme4 and *lmerTest* packages in R) [56], with a random intercept for site phase. This approach partitions the variance into a between-site component, which captures the scaling relationship of interest—how log mean residence size changes across settlements of different sizes—and a within-site component, which captures local heterogeneity in house sizes within individual settlements. The intraclass correlation coefficient (ICC) quantifies the proportion of total variance attributable to differences between site phases. OLS results are reported in Supplementary Materials for comparability with previous scaling studies.

To assess how far the estimated scaling exponent depends on which houses happened to be excavated and measured within each settlement, rather than on the underlying relationship between house and settlement size, we additionally quantified its uncertainty through a within-site bootstrap. Houses were resampled with replacement within each site phase—holding the set of sites and each site’s sample size fixed—and the baseline model was refitted to each of 1000 resampled datasets. From the resulting bootstrap distribution of β we report the mean and a 95% percentile interval alongside the model-based standard error. We further assessed the robustness of the baseline exponent in three ways: to the influence of individual site-phases, through a leave-one-out procedure with formal influence diagnostics (DFBETAS and Cook’s distance); by estimating the exponent within each region; and by refitting the model with each region removed in turn (leave-one-region-out). Full results are reported in Supplementary Materials.

The analytical sequence for the scaling analysis is designed to address the three research questions outlined above. To investigate when scaling relationships emerge, we examine chronological variation across four periods (Neolithic, Chalcolithic, Bronze Age, Iron Age), preceded by a baseline analysis across the entire dataset that tests whether palatial complexes affect results (they do not, and all subsequent analyses include them). To assess the regional specificity of tell-based urban forms, we examine geographical variation across five sub-regions (Anatolia, Cyprus, Levant, Northern and Southern Mesopotamia). To test whether scaling variation is structured by chronology, by geography, or by both independently, we compared three mixed-effects models—a chronology-only model (allowing slopes to vary by period), a geography-only model (allowing slopes to vary by region), and an additive model including both—using likelihood ratio tests, AIC and BIC. [1.1]To investigate the role of institutional complexity in both scaling and inequality, we examine variation by political organisation using Johnson and Earle’s [49] classification, amalgamated into low-complexity (Family-level, Local Polities), mid-complexity (Big Man, Chiefdoms), and high-complexity (Archaic States, Empires). A complete summary of the statistical models used in this study—each model’s specification, the question it addresses, and where its results are reported—is provided in Table 1.

**Table 1:** Overview of statistical models across the three analytical strands (settlement scaling, population density, and inequality).

Analysis	Role	Model specification	Method	Question / hypothesis	Reported in
***Settlement scaling***					
*Baseline scaling*	Primary	log₁₀(house area) ~ log₁₀(site area) + (1|site phase)	Mixed-effects (± palaces); within-site bootstrap	*Is there an overall sub-linear scaling of house size on settlement area?*	Table 2
*OLS comparison*	Robustness	log₁₀(house area) ~ log₁₀(site area)	OLS (± palaces)	*Is the estimate robust to ignoring within-site clustering?*	S1 Table in S1 File
*Aggregation sensitivity.*	Robustness	log₁₀(mean/median house area) ~ log₁₀(site area)	OLS on site-phase means and medians	*Is β robust to the choice of analytical unit?*	S2 Table in S1 File
*Leave-one-site-out.*	Robustness	log₁₀(house area) ~ log₁₀(site area) + (1|site phase), dropping one site-phase	Leave-one-out refit + DFBETAS / Cook’s distance	*Is the baseline exponent driven by any individual site-phase?*	S3 Table; S1 Fig in S1 File
*Chronological scaling*	Primary	log₁₀(house area) ~ log₁₀(site area) + (1|site phase), per period	Mixed-effects per period; within-site bootstrap	*Does β change across periods (when does scaling emerge)?*	Fig 2; S4 Table in S1 File
*Y₀ secular trend*	Derived	Y₀ ~ midpoint date	OLS on per-period intercepts; Wald z-tests	*Does baseline house size (Y₀) change over time independently of scaling?*	S4 Table; S2 Fig in S1 File
*Time vs space comparison*	Primary	log₁₀(house area) ~ log₁₀(site area) × Period + log₁₀(site area) × Region + (1|site phase)	Mixed-effects; LRT / AIC / BIC	*Is β variation structured by chronology, geography, or both?*	Table 3
*Geographical scaling*	Primary	log₁₀(house area) ~ log₁₀(site area) + (1|site phase), per region	Mixed-effects per region	*Does β vary across regions?*	S5 Table; S6 Table in S1 File
*Within-region scaling*	Robustness	log₁₀(house area) ~ log₁₀(site area) + (1|site phase), per region × period cell (≥ 5 site-phases)	Mixed-effects per cell	*Does the emergence of scaling hold within individual regions?*	S7 Table in S1 File
*Leave-one-region-out.*	Robustness	log₁₀(house area) ~ log₁₀(site area) + (1|site phase), dropping one region	Leave-one-region-out refit	*Is the baseline exponent driven by any single region?*	S8 Table in S1 File
*Scaling by political complexity.*	Robustness	log₁₀(house area) ~ log₁₀(site area) + (1|site phase), per polity type/group	Mixed-effects per polity category	*Does β vary systematically with political complexity?*	S9 Table; S3 Fig in S1 File
*Range restriction test*	Robustness	log₁₀(house area) ~ log₁₀(site area) + (1 | site phase); Bronze Age restricted to Neolithic site-size range	Mixed-effects on restricted sample	*Is the Neolithic absence of scaling an artefact of its narrow site-size range?*	Discussion (text)
*Neolithic absence tests*	Robustness	sub-region centering; storage-regime split; vertical-space simulation	Mixed-effects / OLS	*Is the Neolithic absence of scaling an artefact (composition, proxy, or unrecorded upper storeys)?*	S13 Table; S14 Table; S6 Fig in S1 File
***Inequality***					
*Gini–size correlation*	Primary	Gini ~ log₁₀(site area), by polity group × hierarchy position	Pearson correlation	*Does within-site inequality rise with settlement size, and in which polities?*	Table 4; Fig 3
*Scaling residuals vs inequality (ξᵢ–Gini)*	Primary	Gini ~ log₁₀(site area) + ξᵢ	Partial correlation + F-test on baseline BLUPs	*Do scaling residuals (deviations from expected productivity) predict inequality?*	Fig 5
*Dispersion-metric robustness*	Robustness	log-variance and P90/P10 ~ log₁₀(site area), by polity group	Pearson correlation	*Is the Gini–size pattern robust to the choice of dispersion metric?*	S10 Table; S4 Fig in S1 File
*HP threshold sensitivity*	Robustness	Gini–size correlation at HP cut-offs 0.5–0.8	Pearson correlation across thresholds	*Is the “major centre” classification robust to the HP threshold?*	S15 Table in S1 File
***Population density***					
*Density scaling*	Primary	log₁₀(total HH) ~ log₁₀(site area), per period	OLS per period (± residential correction)	*How does household density scale with settlement size in each period?*	Fig 4; S11 Table in S1 File
*Population estimates*	Derived	from density-scaling intercepts	back-transformed intercept × 5 persons/HH	*What population densities do the scaling intercepts imply?*	Table 5
*Residential-correction sensitivity*	Robustness	log₁₀(total HH × res_prop) ~ log₁₀(site area), residential proportion varied over a grid	OLS per period across the assumption grid	*Are the density exponents robust to the residential-correction assumptions?*	S12 Table; S5 Fig in S1 File

Models are grouped by analytical strand. “Role” indicates whether a model is a primary analysis (Primary), a robustness check on a primary model (Robustness), or a quantity derived from a primary model (Derived). Specifications are given in R notation; “(1 | site phase)” denotes a random intercept for site phase. “Reported in” indicates the table(s) and figure(s) where each model’s results appear.

For the chronological analysis, we also report the model intercept for each period. Back-transformed to the original scale (*Y₀* = 10^intercept), this quantity represents the predicted house size at a standardised settlement area of 1 ha (since log₁₀(1) = 0) and provides a measure of baseline house size independent of the scaling relationship. Secular change in Y₀ is assessed through linear regression of the intercept against the mean midpoint date of each period, with pairwise comparisons between consecutive periods using Wald z-tests, in order to determine whether baseline house size changed significantly between periods independently of any scaling relationship with settlement size.

The analysis of inequality employs the Gini coefficient of residence sizes within each site phase [48,57], examined in relation to settlement size and political complexity, as well as the hierarchical position within settlement systems. To assess the role of settlement hierarchy, we use the variables *WhichLevel* (a site’s rank within its settlement system) and *NOfLevels* (the total number of levels in that system), following the coding in Kohler et al. [48]. The GINI Special Feature employs two hierarchy measures: Social Advantage (*SA* = *WhichLevel* + *NOfLevels*), an ordinal estimate of network scale, and a binary Apex/Basal distinction where Apex sites are those at the top of their hierarchy (*WhichLevel* = *NOfLevels*). Because the Apex classification does not capture functionally important centres that occupy the second tier of multi-level systems—such as Kültepe-Kanesh, a major commercial hub at level 3 in a 4-level system, see below—we defined a continuous Hierarchy Position index (*HP* = *WhichLevel* / *NOfLevels*). In our high-complexity sub-sample, Apex sites and sites with *HP* ≥ 0.8 are identical (*N* = 18, all with *HP* = 1.0). At this threshold the Gini-size correlation is not significant. A sensitivity analysis across *HP* thresholds from 0.5 to 0.8 (S15 Table in S1 File) shows that the correlation is robust from 0.5 to 0.7 but breaks down at 0.8, where functionally important centres are misclassified as peripheral. The threshold of *HP* ≥ 0.7 is therefore adopted, as it represents the upper bound at which the distinction holds. This means that in a 3-level system (i.e., one with villages, towns and cities) it captures the top tier (cities, *HP* = 1.0) and excludes the middle (towns, *HP* = 0.67); in a 4-level system (i.e., one with big cities, small cities, towns and villages) it captures the top two tiers (big cities and small cities, *HP* = 0.75 and 1.0). Sites with HP ≥ 0.7 are classified as “major centres” and sites with HP < 0.7 as “minor/peripheral.” Settlement hierarchy analyses appear in Supplementary Materials.

To connect the scaling and inequality analyses, we test whether the residuals of the scaling relationship predict within-site inequality. SST proposes that variation in socioeconomic outcomes across settlements of similar size is captured by the residuals ξᵢ [18], but whether these residuals predict inequality in the distribution of house sizes has not been tested empirically. We extract site-level random intercepts (Best Linear Unbiased Predictors, BLUPs, denoted ξᵢ) from the baseline mixed-effects model. In this analysis, positive values indicate site phases where houses are larger than predicted given settlement area, negative values the inverse. Positive deviations therefore mark settlements with potentially greater surplus available for unequal distribution. We test the correlation between ξᵢ and Gini both overall and controlling for settlement area via partial correlation, and through a multiple regression (Gini ~ log₁₀ *Site Area* + ξᵢ) with an F-test comparing models with and without the residual term. As a robustness check, we compute two further dispersion measures within each site phase: the variance of log₁₀(house area), a distribution-free alternative to the Gini coefficient more directly grounded in the log-normal assumptions of SST, and the P90/P10 ratio of house sizes, based on the tails of the size distribution; both are reported in Supplementary Materials.

Beyond the scaling analysis described above, our dataset also allows us to address a long-standing question in the archaeology of Southwest Asia: how many people lived in these settlements? Population estimates for Ancient Southwest Asia have varied widely, with most surveys adopting uniform coefficients of 100–200 persons per hectare across settlements that differ by orders of magnitude in size [29,45]. More recent work has sought to refine these estimates by propagating uncertainty through wide envelopes such as the range of 248–1205 persons per hectare proposed by Postgate [43] for Abu Salabikh, or by differentiating coefficients by site type or function [58,59]. The limitations of these conventions have been recognised for some time: applying a single coefficient to settlements that vary by three orders of magnitude in area assumes a constant density that is unlikely to hold [60,61], particularly because the residential proportion of a site is itself highly variable across periods and morphologies, as shown by Stone [44] for Bronze Age Mesopotamia and by Kuijt and Marciniak [62] for the Neolithic. All such approaches, however, postulate a density coefficient a priori rather than deriving the area-population relationship from the data themselves.

SST offers an alternative route to this problem. Because the theory predicts how aggregate quantities scale with population, the intercepts of the scaling relationships can be read directly as estimates of baseline density at a standardised settlement size. Specifically, the relationship between the total number of households and settlement area should follow *N* ∝ *A*^(2/3) when settlements are spatially amorphous, or *N* ∝ *A*^(5/6) when infrastructure networks structure the built environment [6,8], and the back-transformed intercept of this relationship gives the expected number of households at *A* = 1 ha. This approach makes no prior assumption about a uniform density coefficient, but instead it allows density to vary systematically with settlement size, as predicted by the theory and as recognised in qualitative form by previous archaeological work.

To implement this analysis, we extrapolated household counts from the excavated window to the whole site for each site phase, following *TotalHH* = (*WindowHH* / *WindowArea*) × *TotalArea*, where *WindowHH* and *WindowArea* refer to the number of measured houses and the spatial extent of the excavated area respectively, and *TotalArea* is the phase-specific settlement size. We excluded the Palaeolithic, for which the number of site phases is insufficient to support reliable extrapolation, and the site of Kerkenes Dağ, whose house data derive from geophysical survey rather than excavation [63].

For each period we fitted log₁₀(*TotalHH*) = α + β × log₁₀(*TotalArea*) by ordinary least squares, treating each site phase as a single observation since the analysis operates at the aggregate level. The back-transformed intercept (10^α) gives the predicted number of households per hectare at a standardised settlement area of 1 ha. To correct for the observation that the residential proportion of a site is not constant but decreases with site size, as documented by Stone [44] for Bronze Age Mesopotamia and by Kuijt and Marciniak [62] for Neolithic settlements, we also fitted a residential-corrected version in which household counts are scaled by a proportion that decreases log-linearly from 0.90 at 1 ha to 0.60 at 100 ha for the Chalcolithic, Bronze Age, and Iron Age; for the Neolithic, where the dispersed character of large settlements is already captured by the window-based estimate, no correction is applied. To verify that the corrected exponents do not depend on these two assumed endpoints, we varied both independently over a grid from 0.50 to 1.00 and refitted the per-period model for every combination (S12 Table; S5 Fig in S1 File). For comparability with the existing literature on Mesopotamian population estimates, we also convert household density to persons per hectare using a multiplier of 5 persons per household, justified in Results.

All analyses were conducted in R v. 4.4.0 [64] using the *lme4* [56] and *lmerTest* packages [65] for mixed-effects models, *MuMIn* for model diagnostics [66], *DescTools* for Gini coefficients [67], and *influence.ME* for the leave-one-out influence diagnostics [68]. The dataset, together with the R scripts used to generate all results reported in this study, and an executable notebook that reproduces the full analysis are available in the Zenodo repository (https://doi.org/10.5281/zenodo.20172243).

## Results

Across the full dataset, the baseline analysis reveals a significant sub-linear scaling relationship between house size and settlement area. The mixed-effects model estimates β = 0.298 when all structures are included and β = 0.286 when palaces are excluded (Table 2), both highly significant. The difference between the two is negligible (Δβ = 0.012), meaning that palatial complexes do not drive the scaling relationship, therefore all subsequent analyses retain them. OLS results and sensitivity analyses based on site-phase means and medians are reported in Supplementary Materials (S1 and S2 Tables in S1 File) and confirm these estimates across alternative statistical approaches. A within-site bootstrap (1000 replicates) yields a mean exponent identical to the model estimate (β = 0.297, 95% CI [0.277, 0.317]; Table 2), confirming that the baseline relationship does not depend on which houses happened to be excavated and measured within individual site-phases. A complementary leave-one-out analysis, refitting the baseline model with each site-phase omitted in turn, confirms that no single site-phase drives the relationship: the exponent ranges from 0.282 to 0.308 across all refits (full-sample β = 0.298), and the largest change produced by removing any one site-phase is Δβ = 0.015 (S3 Table; S1 Fig in S1 File). The exponent is likewise robust to regional composition: refitting the baseline with each region removed in turn leaves it within the range 0.272–0.335 (full-sample β = 0.298), the largest shift following removal of Northern Mesopotamia (S8 Table in S1 File).

**Table 2:** Baseline scaling (mixed-effects model).

Metric	With palaces	Without palaces	Difference
β (scaling)	0.298	0.286	0.012
SE	0.038	0.038	—
df (Satterthwaite)	102.6	103.0	—
*R*² marginal	0.279	0.277	0.001
*R*² conditional	0.661	0.690	−0.029
ICC	0.530	0.571	−0.041
*N* buildings	1852	1841	−11 (palaces)
*N* sites	80	80	0
*N* site-phases	107	107	0
*p* value	< 0.001	< 0.001	—
Δβ test			*t* = 0.226, *p* = 0.821 (n.s.)
β boostrap mean	0.297	0.285	—
β boostrap SE	0.011	0.010	—
β boostrap 95 CI	[0.277, 0.317]	[0.267, 0.305]	—

Linear mixed-effects model with random intercept for site phase. *R*^2^ marginal = variance explained by site area (fixed effect); *R*^2^ conditional = variance explained by site area and site-phase identity (fixed + random effects); ICC = proportion of total variance attributable to between site-phase differences. OLS results reported in Supplementary Materials.

More than half of the total variance in house size is attributable to differences between site phases rather than to variation among buildings within the same phase (ICC = 0.53). Local factors, including construction traditions, available materials, and site-specific socioeconomic conditions, clearly exert a strong influence on house size, one that operates alongside, and is at least as powerful as, the general scaling relationship with settlement size. Marginal R^2^ (variance explained by site area alone) is consistent with the OLS estimates; conditional R^2^ (site area plus site-phase identity) accounts for approximately two-thirds of total variance.

The observed baseline β of c. 0.30 can be compared with the theoretical expectation. As discussed in the Introduction, average house size is an intensive quantity that SST predicts should scale with population at approximately 1/6. Given the substitution of settlement area for population (*A* ∝ *N*^2/3), this translates to an expected exponent of approximately 1/4 (0.25) for house size against site area. Our baseline estimate of 0.30 falls close to this adjusted prediction, slightly above but within the range consistent with sub-linear scaling of an intensive residential quantity. Other archaeological studies measuring comparable intensive quantities against population report lower exponents that cluster around the predicted 1/6: mean domestic mound area in the Basin of Mexico (β = 0.19) [7], mean house area among Mandan/Hidatsa villages (β = 0.16) [17], and domestic structure size in the Peruvian Andes (β = 0.13) [13]. The difference from our estimate is expected, since these studies use population directly as the independent variable. The closest regional comparison is Squitieri and Altaweel [37], who report β = 0.24 for their pre-imperial period (c. 3000–800 BCE) and β = 0.35–0.41 for the Age of Empires (c. 800 BCE – 224 CE). Our baseline sits between these values which is not surprising given that our dataset spans both periods but is weighted towards earlier and smaller settlements.

The consistency of this pattern across both statistical approaches and methodological decisions regarding palace inclusion establishes a solid foundation for examining whether this sub-linear scaling varies by chronological period, geographical region, or political unit type.

**Fig 2:**
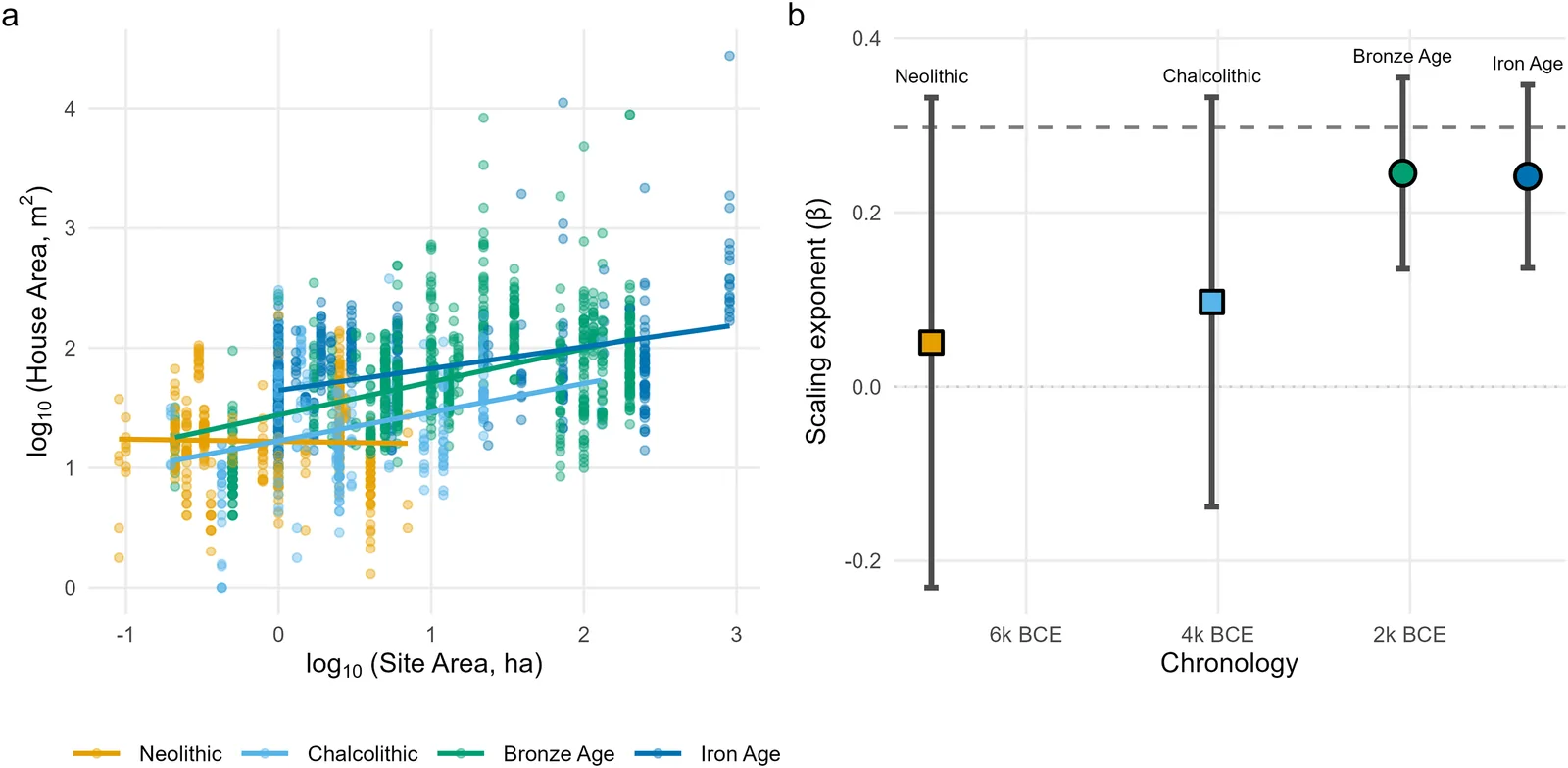
Chronological variation in scaling relationships between residence size and settlement size. (a) Log-log scatter plot of individual building areas against site areas, coloured by period, with linear regression lines showing the scaling relationship for each period. (b) Scaling exponents (β) with 95% confidence intervals from mixed-effects models, positioned at the mean midpoint date of each period. The dashed line indicates the overall baseline β (0.298); the dotted line marks β = 0. Filled circles denote statistically significant relationships (*p* < 0.05); filled squares denote non-significant relationships.

The chronological analysis (Fig 2; S4 Table in S1 File) reveals a clear trajectory in scaling relationships across Southwest Asian settlement history. During the Neolithic and Chalcolithic, house size bears no significant relationship to settlement size (β = 0.051, *p* = 0.727 and β = 0.097, *p* = 0.427, respectively). In both periods, house sizes are shaped primarily by local, site-specific factors. Between 50% and 74% of the variance lies between site phases (ICC), rather than within them.

Significant scaling first appears during the Bronze Age (β = 0.246, *p* < 0.001), with houses growing at roughly one-quarter the rate of settlement expansion. The Iron Age maintains the same relationship (β = 0.242, *p* < 0.001), suggesting that once scaling emerges it persists across different political configurations. Note, however, that the absence of measurable scaling in the Chalcolithic period may partly reflect the compositional limitations of the available samples. The Chalcolithic sample is dominated by small Anatolian settlements (8 sites, 111 buildings, all under 6 ha) and Cypriot villages (4 sites, 65 buildings). Chalcolithic Anatolian communities remained organised as increasingly complex but pre-state societies that had not reached proto-urban scales [69,70], while Chalcolithic Cyprus was a decidedly pre-urban society and social differentiation, though present, did not consolidate into persistent inequalities [71]. Three of the four Mesopotamian sites included in the dataset are from the north (Habuba Kebira South, Tell Brak, and Tepe Gawra), with the southern alluvial heartland where the earliest cities developed represented by a single site (Tell Abada). Only Tell Brak (130 ha), in northern Mesopotamia, can be considered urban in scale, and with only five houses, it contributes minimally to the regression.

An interesting result of the chronological analysis is that every individual period yields β values lower than the overall baseline (β = 0.298), a phenomenon known as Simpson’s Paradox. As Bettencourt et al. [46] and Lobo et al. [18] note, prefactors of scaling relationships change over time through processes independent of population growth. In this sense the aggregate β, estimated across all periods, conflates these shifting prefactors with the within-period scaling relationship, producing an exponent that is higher than any single period estimate.

The model intercepts make this long-term shift visible in quantitative terms. Back-transformed to the original scale, the prefactor *Y*₀—the predicted house size at a standardised settlement area of 1 ha—increases regularly from 16.9 m^2^ in the Neolithic to 23.0 m^2^ in the Chalcolithic, 31.8 m^2^ in the Bronze Age, and 41.8 m^2^ in the Iron Age (S4 Table; S2 Fig in S1 File). The trend is linear (*R*^2^ = 0.98, *p* = 0.012), suggesting that baseline house size more than doubled over time independently of changes in the scaling exponent. No single consecutive period transition is individually significant (all *p* > 0.2), but the cumulative trajectory is clear. What is perhaps more interesting is that *Y*₀ rises at a comparable rate during the Neolithic-Chalcolithic interval, when β ≈ 0.07 and non-significant, to that during the Bronze-Iron Age interval, when β ≈ 0.24 and highly significant, suggesting that the growth in baseline house size reflects processes (e.g., expanding material resources, improving construction technologies, increasing household investment capacity and labour) that operate independently of agglomeration scaling. This result implies that the long-term improvement in residential investment across Southwest Asia was not contingent on the emergence of urban-scale agglomeration. Houses grew larger over the millennia regardless of whether settlement size predicted their dimensions. This pattern is consistent with the technology-driven growth identified by Ortman and Lobo [47], who use SST to decompose overall economic growth into agglomeration-driven (’Smithian’) and technology-driven components. The steady rise in *Y*₀ across our sequence suggests that improving material technologies and household capacities drove baseline residential investment independently of changes in settlement size or the emergence of agglomeration effects. We return to this distinction in the Discussion.

From a geographical point of view, scaling exponents vary substantially, from β = 0.443 in the Levant to β = 0.180 in northern Mesopotamia, with Cyprus showing no significant scaling (S5 Table in S1 File). These differences, however, are difficult to read in isolation because each region’s sample has a markedly different chronological composition: the Levant is dominated by Bronze and Iron Age sites, Anatolia spans all four periods more evenly, and southern Mesopotamia—where the earliest cities emerged—is represented by only 9 sites, none earlier than the Late Chalcolithic (S6 Table in S1 File). Estimating scaling within individual region × period cells confirms that this geographical structure does not undermine the overall relationship: the chronological emergence of scaling holds within regions and not only across them, being absent in the Neolithic and Chalcolithic and significant in the Bronze Age Levant (β = 0.725, p = 0.005) and Northern Mesopotamia (β = 0.210, p = 0.011) (S7 Table in S1 File).

The relative contributions of time and space can be disentangled by comparing the chronology-only, geography-only, and additive models described in the Methods (Table 3). Individually, the two dimensions explain similar amounts of variance, but adding geography to the chronology-only model significantly improves fit (*χ*² = 32.0, *p* < 0.001), as does adding chronology to the geography-only model (*χ*² = 22.7, *p* < 0.001), indicating that temporal and spatial variation structure scaling independently. This result reflects the fact that urbanisation trajectories differed across the study region: the same period label encompasses different levels of social and economic integration in different regions. The regional ICC values reinforce this point: in Mesopotamia, where urban economies generated substantial intra-settlement differentiation, ICC values are low (0.23–0.25), meaning most variation in house size occurs within sites; by contrast, Cyprus and the Levant show high ICC values (0.65–0.72), indicating internally homogeneous communities that differ primarily from one another. We return to the theoretical implications of these patterns in the Discussion.

**Table 3:** Model comparison: Chronology, geography, and additive models.

Model	Fixed effects	*k*	AIC	BIC	*R*² marg.	*R*² cond.	*χ*²	df	*p*
Chronology only	site area × period	10	1075.2	1130.3	0.344	0.649	—	—	—
Geography only	site area × region	12	1075.8	1142.0	0.361	0.646	—	—	—
Additive (both)	site area × period + site area × region	18	1084.1	1183.4	0.414	0.652	—	—	—
Chrono → Additive							32.0	8	< 0.001
Geo → Additive							22.7	6	< 0.001

All models are linear mixed-effects with random intercept for site-phase, fitted to 1,838 buildings (105 site-phases) after excluding the Palaeolithic. *k* = total estimated parameters (fixed effects + variance components). AIC and BIC computed via maximum likelihood refit. *R*^2^ marginal = variance explained by fixed effects; *R*^2^ conditional = variance explained by fixed and random effects combined. Likelihood ratio tests (*χ*²) assess whether adding one set of predictors significantly improves the model already containing the other.

Having established how scaling relationships between house size and settlement area vary across time and space, we now turn to a distinct but related question: how the distribution of house sizes within settlements—a measure of residential wealth inequality—relates to settlement size and political organisation. While scaling exponents do not vary systematically with political complexity (S9 Table; S3 Fig in S1 File), the relationship between settlement size and within-site inequality reveals a clearer pattern. We calculated the Gini coefficient and Hierarchy Position index as defined in the Methods, where sites classified as major centres include capitals and primary regional centres including Babylon and Assur, as well as the commercial hub of Kültepe-Kanesh.

**Fig 3:**
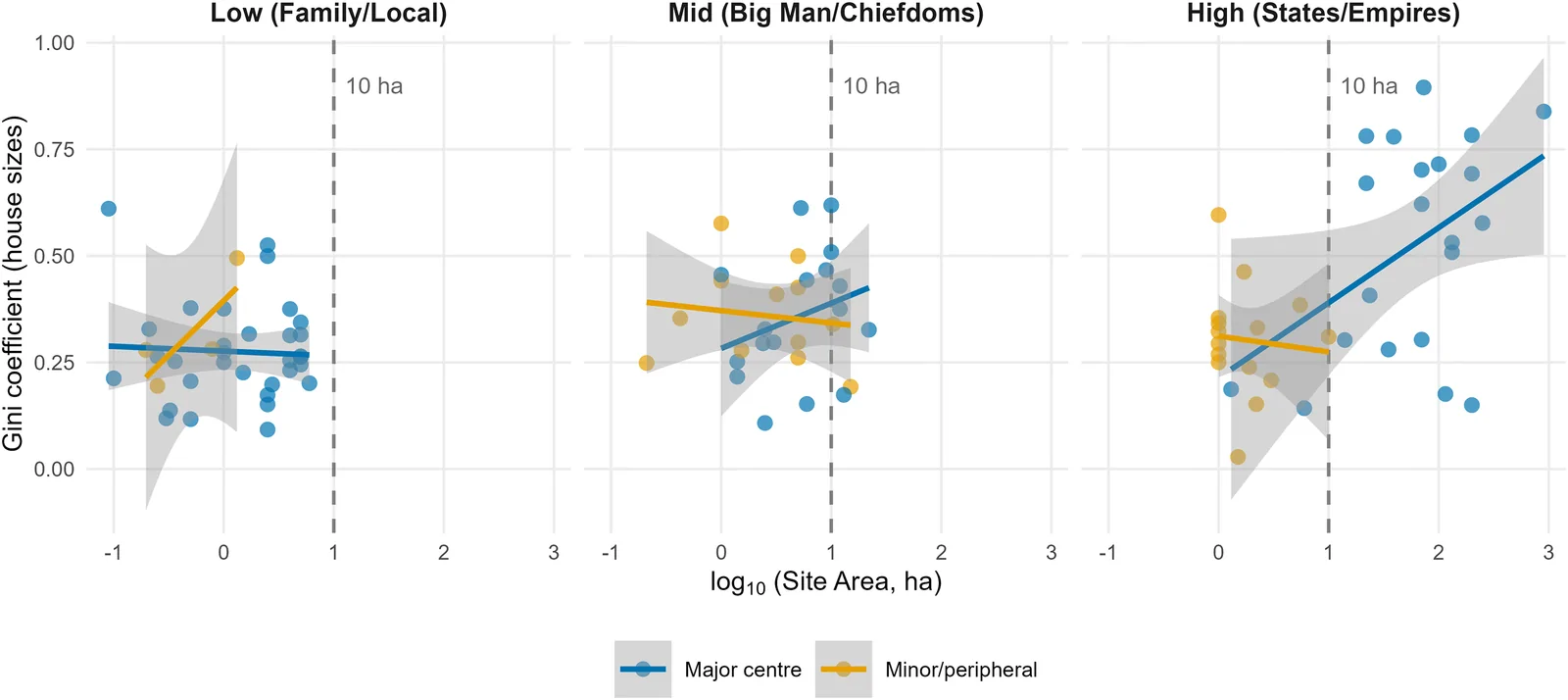
Gini coefficient of house sizes plotted against settlement area (log₁₀), faceted by polity group. Points are coloured by hierarchy position: major centres (*HP* ≥ 0.7, dark brown) and minor/peripheral sites (*HP* < 0.7, light blue). Lines represent linear fits with 95% confidence envelopes. The dashed vertical line marks 10 ha.

Across the full dataset, the Gini coefficient correlates positively with settlement size (*r* = 0.493, *p* < 0.001): larger sites exhibit greater house size inequality. However, this relationship seems to be entirely driven by politically complex systems. In fact, in low-complexity polities (Family-level and Local Polities) mean Gini is low (0.279) and uncorrelated with site area (*r* = −0.019, n.s.). Mid-complexity polities (Big Man and Chiefdoms) show slightly higher inequality (mean Gini = 0.358) but likewise no significant correlation with settlement size (r = 0.088, n.s.). Only in high-complexity polities (Archaic States and Empires) does a strong and significant relationship emerge (*r* = 0.570, *p* < 0.001; Table 4; Fig 3).

**Table 4:** Within-site inequality (Gini coefficient of house sizes) by polity group and hierarchy position.

Polity group	*N* site-phases	*N* sites	Gini mean (SD)	*r* vs site area	*p*
Low (Family/Local)	36	28	0.279 (0.117)	−0.019	0.912
Mid (Big Man/Chiefdoms)	29	22	0.358 (0.135)	0.088	0.649
High (States/Empires)	36	28	0.433 (0.235)	0.570	< 0.001
Major centres (*HP* ≥ 0.7)	21	15	0.526	0.439	0.047
Minor/peripheral (*HP* < 0.7)	15	9	0.303	−0.087	0.758

*r* = Pearson correlation between Gini and log₁₀ site area.

Within high-complexity systems, a threshold effect is apparent at approximately 10 ha: sites below 10 ha have a mean Gini of 0.285, comparable to low-complexity settlements, whereas sites above 10 ha reach a mean Gini of 0.551. This threshold has been used as a heuristic tool to distinguish sites functioning as urban centres from smaller dependent settlements [29,72,73]. The distinction between major centres and minor/peripheral sites further clarifies this pattern. Among high-complexity polities, major centres show a significant positive correlation between Gini and settlement size (*r* = 0.439, *p* = 0.047), ranging from small centres like Tell el-Far’ah North (6 ha, Gini = 0.143) to Babylon (900 ha, Gini = 0.839). Minor or more peripheral sites, by contrast, show no such relationship and maintain uniformly low inequality (mean Gini = 0.303) regardless of site size.

Finally, we examine whether scaling residuals predict inequality. Based on our dataset, they do not. Site-level BLUPs (ξᵢ) from the baseline mixed-effects model, which captures whether houses at a given site are larger or smaller than predicted by the scaling relationship, show no significant correlation with Gini coefficients, either overall (*r* = −0.053, *p* = 0.597) or controlling for settlement area (partial *r* = −0.077, *p* = 0.446). Adding ξᵢ to a regression of Gini on settlement area does not improve model fit (*F* = 0.59, *p* = 0.446). The null holds across all three polity groups. It should be noted that BLUPs from mixed-effects models tend to shrink toward the population mean, particularly where there are small numbers of observations, which could attenuate any underlying correlation. However, only 12% of site phases in our dataset have six or fewer measured buildings, while 72% have ten or more, suggesting that shrinkage is unlikely to substantially affect these results. For this reason, we are confident to state that inequality appears to be associated with settlement scale itself, not with a site’s deviation from the expected scaling relationship. As robustness checks, we also computed the variance of log₁₀(house area) and the P90/P10 ratio of house sizes. Both reproduce the Gini pattern, correlating with settlement size only in high-complexity polities, although the relationship reaches significance for the log-variance and only approaches it for the more sampling-sensitive P90/P10 ratio (S4 Fig; S10 Table in S1 File).

**Fig 4:**
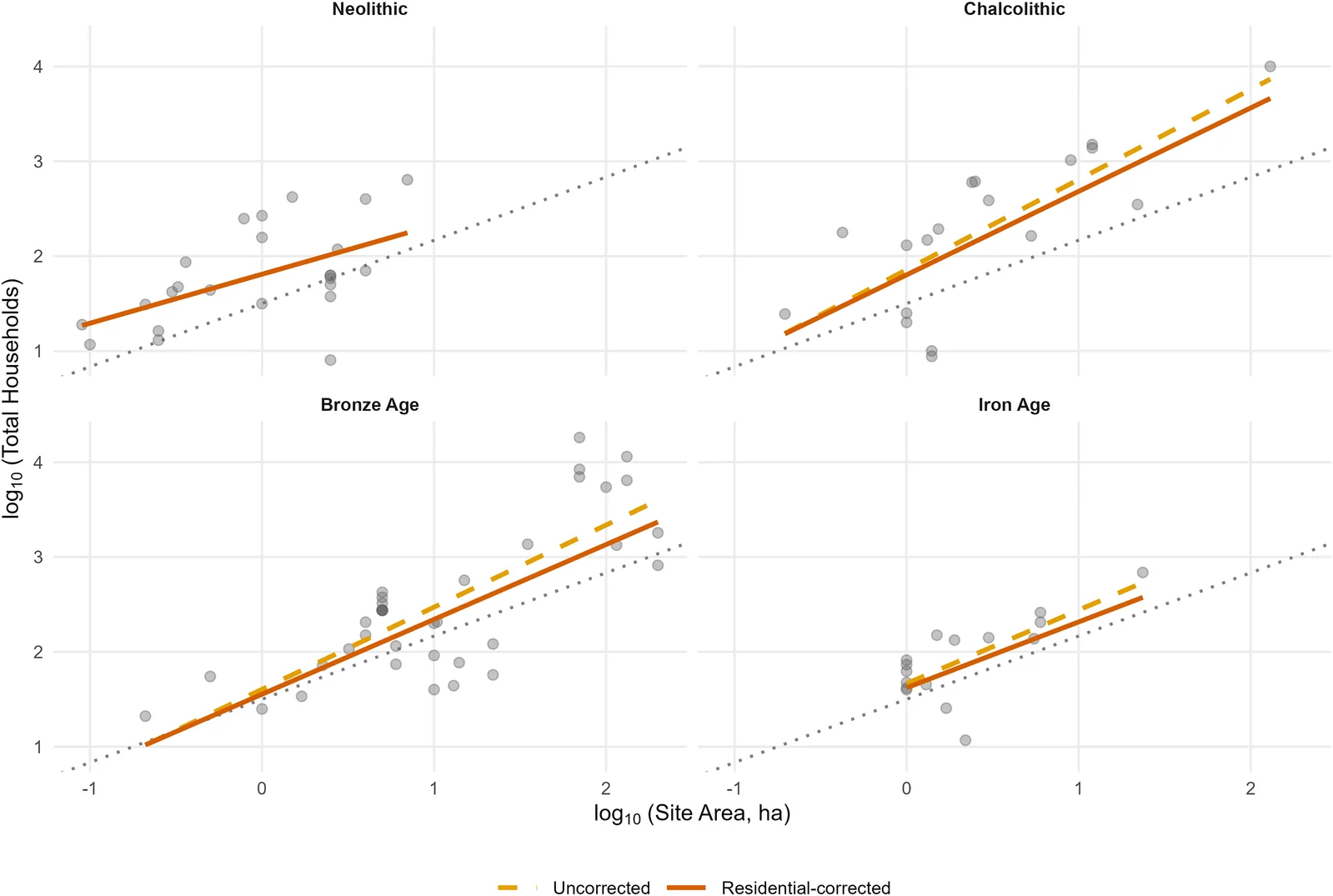
Density scaling per period. Each panel shows individual site phases (grey points), the uncorrected regression line (dashed) and the residential-corrected regression line (solid). The dotted line marks the SST prediction of β = 2/3 for amorphous settlements as a reference.

We conclude the Results by addressing the population-density question raised in the Methods. Across the four periods of our dataset, fitting log₁₀(*TotalHH*) = α + β × log₁₀(*TotalArea*) by ordinary least squares yields significant scaling relationships in all cases (Fig 4; S11 Table in S1 File). The uncorrected scaling exponents are β = 0.52 (*p* = 0.006) for the Neolithic, 0.95 (*p* < 0.001) for the Chalcolithic, 0.87 (*p* < 0.001) for the Bronze Age, and 0.78 (*p* = 0.003) for the Iron Age. After applying the residential correction (see Methods), the scaling exponents are β = 0.52 for the Neolithic (unchanged), 0.88 for the Chalcolithic, 0.79 for the Bronze Age, and 0.69 for the Iron Age. These corrected exponents are robust to the choice of residential-proportion endpoints: across the full grid of assumptions the exponent shifts by no more than 0.13 within any period, and each period remains within the same SST regime, so the classifications discussed below are not artefacts of the specific correction values adopted (S12 Table; S5 Fig in S1 File).

The back-transformed intercepts of the residential-corrected regressions give the predicted number of households per hectare at a standardised settlement area of 1 ha: 64 (Neolithic), 63 (Chalcolithic), 36 (Bronze Age), and 42 (Iron Age). To convert these household densities into population densities, we adopt a constant multiplier of 5 persons per household. This value sits within the range of textual estimates available for Ancient Mesopotamia, for example 5.29 persons per household at Kassite Nippur [74], 4.64 at Old Babylonian Kish [75], and is consistent with the broader range of 4.4–7 proposed by Stager [76] on the basis of life-expectancy and fertility considerations.

Population estimates derived from the residential-corrected scaling at three representative settlement sizes are reported in Table 5. For a 10 ha Bronze Age settlement the model predicts approximately 1100 inhabitants at a density of 110 persons per hectare; for a 100 ha Bronze Age site, approximately 6760 inhabitants at 68 persons per hectare; for a 100 ha Iron Age site, approximately 5100 inhabitants at 51 persons per hectare; and for a 100 ha Chalcolithic site (a value approached in our sample only by Tell Brak at 130 ha), approximately 18,000 inhabitants at 182 persons per hectare.

**Table 5:** Estimated total population and density at representative settlement sizes, by period.

Period	1 ha	10 ha	100 ha
Neolithic	320 (320)	1060 (106)	3485 (35)
Chalcolithic	315 (315)	2405 (240)	18,240 (182)
Bronze Age	180 (180)	1100 (110)	6760 (68)
Iron Age	210 (210)	1035 (104)	5105 (51)

Values shown as total population (persons per hectare in parentheses), based on residential-corrected density scaling and a multiplier of 5 persons per household.

## Discussion

This study presents the first multi-phase analysis of settlement scaling relationships across Ancient Southwest Asia, covering over twelve millennia from the Late Epipalaeolithic to the Iron Age. Three principal findings emerge. First, scaling relationships are not uniformly present but emerge historically. Second, where they emerge, their magnitude varies across both time and space but is generally consistent with SST predictions. Third, the relationship between scaling and inequality is institutionally contingent.

Scaling relationships between house size and settlement size are not uniformly present across the Southwest Asian record. No significant relationship exists during the Neolithic, scaling becomes detectable only from the Bronze Age onwards and persists through the Iron Age at a comparable magnitude. SST does not predict that scaling will be found wherever one looks, but it specifies the conditions under which agglomeration effects should arise (i.e., strongly mixing networks operating within bounded spatial containers) [18] and predicts the parameter values when those conditions are met. The absence of scaling in the Neolithic is thus diagnostic rather than anomalous. It indicates that these settlements did not yet function as the kind of integrated interaction networks that SST presupposes, analogously to the dispersed Maya case documented by Smith et al. [11]. A potential concern here is that the limited range of settlement sizes in the Neolithic sample (c. 0.1−7 ha) may reduce statistical power to detect scaling even if present, since the confidence intervals for the Neolithic β (−0.23 to 0.33) encompass both meaningful scaling and its absence. To address this, we restricted the Bronze Age sample to the same site-area range as the Neolithic. Under these conditions, the Bronze Age scaling remains significant in the restricted sample (β = 0.381, *p* = 0.001, *N* = 19 site-phases), indicating that the absence of Neolithic scaling is not simply an artefact of the size range available.

The Chalcolithic presents a more ambiguous picture because our sample for this period is dominated by small Anatolian and Cypriot communities that had generally not reached proto-urban scales, while the major centres of Late Chalcolithic Mesopotamia—where scaling might be expected—are either absent from the sample or contribute too few buildings to influence the regression. Whether scaling was genuinely absent in the Chalcolithic or simply undetectable given the compositional limitations of our sample remains a statistically open question based on the available dataset. To resolve this we need to expand domestic exposures during this period across the region. Independently of these changes in the scaling exponent, baseline house size—captured by the model prefactor *Y*₀—more than doubled from the Neolithic to the Iron Age, documenting a growth in household residential capacity that continued whether or not agglomeration scaling was active.

Second, once scaling emerges, the observed exponent (β ≈ 0.30) is close to the theoretical prediction for an intensive quantity regressed against settlement area (≈ 1/4) [6], and consistent with values reported for comparable variables in Mesoamerica (β = 0.13–0.19) [7,13] once the transformation from population to area as independent variable is taken into account. Within the Southwest Asian record, our estimate falls between the pre-imperial (β = 0.24) and imperial-period values (β = 0.35–0.41) reported by Squitieri and Altaweel [37]. Rather than representing an anomaly, this regional consistency points to a distinctive mode of urban organisation shaped by the compact, tell-based settlement tradition of Southwest Asia.

Third, while scaling exponents do not vary systematically with political complexity, within-site inequality—measured through Gini coefficients of house sizes—reveals a sharply contingent pattern. The positive correlation between Gini and settlement size is confined to high-complexity polities and, within these, to major centres occupying the upper tiers of settlement hierarchies. In low- and mid-complexity polities, inequality bears no relationship to settlement size. Basri and Lawrence [38], working with a smaller subset of the same regional dataset, documented a positive relationship between house size inequality and settlement size across the Southwest Asian record as a whole, and observed that the contribution of site size to inequality increased from the Bronze to the Iron Age, with urban centres becoming more unequal while rural sites became more homogeneous. The present analysis, drawing on a substantially expanded dataset and disaggregating by political complexity and hierarchy position, reveals that this aggregate pattern is not a general property of settlement growth. The Gini-size correlation emerges only within politically complex systems and, within these, only at major centres, precisely the sites where institutional mechanisms for surplus concentration and redistribution are in place. The increasing divergence between urban and rural inequality that Basri and Lawrence identified for the Iron Age thus reflects the institutional conditions under which agglomeration translates into residential disparity, rather than a mechanical effect of settlement scale per se. At the same time, the scaling residuals (ξᵢ) do not predict inequality either. In other words, it is settlement scale itself, not a site’s deviation from the scaling relationship, that is associated with greater residential dispersion. Ortman et al. [77] recently demonstrate that agglomeration and productivity account for only a small fraction of observed variance in inequality across the archaeological record. Our results identify one possible source of this unexplained variance in Ancient Southwest Asia. Here, the agglomeration effects that generate residential inequality are institutionally contingent, requiring both political complexity and a site’s functional integration as an administrative or commercial centre. In this perspective, settlement size alone is not sufficient to generate inequality, the institutional apparatus that governs how the returns from agglomeration are distributed among households is what determines whether larger settlements are also more unequal [21,78].

### The emergence of scaling and Smithian growth

The absence of scaling in earlier periods and its emergence in the Bronze Age raises the question of what conditions drove this transition. As noted in the Results, all within-period β values are lower than the overall baseline (β = 0.298), an instance of Simpson’s Paradox produced by the secular increase in both settlement size and house size across periods [18,46]. This carries a methodological warning: pooling data across periods without accounting for temporal structure can produce inflated scaling exponents that do not reflect agglomeration processes operating within any single period. This caveat extends to our own analysis, where each chronological period necessarily pools sites from different regions that were not strictly contemporary. Our within-period β values are therefore best interpreted as averages across diverse settlement systems rather than as parameters of any single contemporaneous network.

The prefactor *Y*₀ quantifies this long-term trend directly. Baseline residence size at a standardised settlement area of 1 ha increased monotonically from 16.9 m^2^ in the Neolithic to 41.8 m^2^ in the Iron Age, which is a more than twofold increase that proceeded at a comparable rate regardless of whether significant scaling was present. This pattern can be situated within the framework for distinguishing sources of economic growth developed by Ortman and Lobo [47]. In their analysis of the pre-Hispanic Northern Rio Grande, the prefactor *Y*₀ remained effectively constant over time, and all improvements in material living standards were attributable to increases in average settlement size, agglomeration-driven (or ‘Smithian’) growth in the strict sense. In our Southwest Asian data, by contrast, *Y*₀ itself increases substantially over time, indicating that baseline productivity grew through processes independent of agglomeration. This component corresponds to what Ortman and Lobo [47] term ‘technology-driven growth,’ related to change in the energetics of social interaction captured by *Y*₀, rather than to Smithian growth proper, which operates through ⟨*N*⟩. The two cases are thus complementary, in that the Northern Rio Grande illustrates pure Smithian growth with stable technology, while Southwest Asia exhibits secular growth in baseline productivity that proceeded whether or not agglomeration scaling was operative. Our data do not identify the specific mechanisms underlying this secular increase in Y₀, but plausible candidates include improvements in construction technology, expanding access to materials, increasing household investment capacity, and the iterative cycles of trade expansion, labour specialisation, and import substitution that Algaze [79] identifies as drivers of economic growth in Ancient Mesopotamian cities. It should also be noted that the growth in Y₀ need not reflect processes entirely independent of urbanisation. If urban centres function as nodes of innovation and exchange whose effects propagate through regional economic networks, the baseline productivity of smaller settlements may rise as an indirect consequence of agglomeration elsewhere in the system—a form of trickle-down transformation that would register as growth in *Y*₀ rather than as within-settlement scaling. The fact that Y₀ grows at a comparable rate during periods with and without significant scaling raises the possibility of decomposing overall growth into its technology-driven and agglomeration-driven components, following Ortman and Lobo [47]. However, their decomposition requires tracking a coherent settlement system through successive periods. Our sub-regions could in principle serve as such systems, but no sub-region in our dataset has sufficient sites in consecutive periods on both sides of the scaling transition to support a reliable decomposition. This remains a promising direction for future research as more data becomes available.

The transition from non-scaling to scaling in our data occurs between the Chalcolithic and the Bronze Age, and by the latter urban forms had consolidated across Mesopotamia, the Levant, and Anatolia. Whether the onset of scaling coincided with the earliest urbanisation of the fourth millennium BCE—when settlements such as Tell Brak and Uruk first expanded beyond 100 hectares [25,33,80]—cannot be determined from our sample, given the compositional limitations of the Chalcolithic dataset discussed above. What our data do show is that the Neolithic, with its well-represented sample, exhibits no scaling, consistent with the broader finding that the transition to agriculture did not in itself generate measurable increases in residential wealth disparities, which remained low for centuries after the Neolithic transitions across multiple world regions [81]. This finding apparently contrasts with Ortman and Coffey’s [17] demonstration that scaling phenomena are detectable even in middle-range societies lacking cities and markets, such as the Central Mesa Verde and Middle Missouri village systems. However, the analytical setting is quite different: in those cases, settlements were contemporaneous components of a single cultural system with shared institutional frameworks, precisely the conditions of social mixing that SST identifies as necessary for agglomeration effects. Our dataset, as noted in the Methods, is structured differently: it amalgamates sites from different sub-regions that were not necessarily contemporaneous and did not form parts of a single interacting system. Detecting scaling under these conditions requires a stronger empirical signal: agglomeration effects must be sufficiently pervasive across the constituent sites of a period for the underlying relationship to survive the pooling of heterogeneous regional trajectories. The contrast between our Neolithic and Bronze results, can be read in this light. The Bronze Age results indicate that this condition is met: scaling remains detectable despite the amalgamation of sub-regional sequences, showing that pooling is not in itself an obstacle to recovering agglomeration signals when those signals are pervasive. In the Neolithic case, by contrast, scaling is absent not because our analytical design has obscured a relationship that was locally present, but for substantive reasons.

These reasons can be examined through three substantive hypotheses, but they need to be set against a key feature of the Neolithic context: whether house size functions there as the same proxy as in later periods. The SST interpretation of average house size as a measure of household productivity presupposes that the house corresponds to a discrete residential group functioning as a basal unit of production and consumption [7]. In Neolithic Southwest Asia, however, the household had not yet crystallised as an autonomous economic unit: storage was often communal, co-residential groups were larger and more variable in composition, and the boundaries between domestic and communal spaces were less clearly defined [82–85]. Against this background, three substantive hypotheses can be considered to explain the absence of Neolithic scaling: (i) that the available data systematically underrepresent vertical architecture, since Neolithic upper storeys are rarely preserved or explicitly documented; (ii) that house size in the Neolithic does not function as a proxy for household productivity in the same way as in later periods; and (iii) that the Neolithic sample aggregates regions with different subsistence baselines, producing artefactually low exponents through compositional effects across the size distribution.

Empirical tests of these three hypotheses yield different results. The third hypothesis is excluded (S6 Fig in S1 File), since centering the data by sub-region and by sub-region × period leaves the Neolithic exponent non-significant under all centering schemes, indicating that the absence of scaling is not an artefact of mixing systems with different baselines. The second hypothesis—that house size in the Neolithic does not function as a proxy for household productivity in the same as in later periods—rests on the framing developed in the introduction, where house size is treated as a measure of the medium-term productive capacity of the residential group. In the Neolithic sample, however, residential groups are not internally homogeneous in this respect: some sites organise storage and production communally, others privately within the house. We tested whether this heterogeneity is reflected in the proxy by partitioning the Neolithic sample by storage regime. The intercept of the residence-size relationship more than doubles between communal-storage sites (*Y*₀ = 12 m^2^) and private-storage sites (*Y*₀ = 30.8 m^2^; S13 Table in S1 File), confirming that house size in the Neolithic captures different productive units in different sites. In other words, house size in the Neolithic tracks primarily the storage regime and therefore cannot relate to settlement scale as a single and unitary proxy in the way SST applications in later periods assume. The first hypothesis is also supported (S14 Table in S1 File), in a way that connects directly to the second. Recent work in urban scaling has shown that average building height scales with population with an exponent of approximately 0.33 [86,87], with the implication that multi-storey construction should be more frequent in larger settlements. If upper storeys in larger Neolithic sites are systematically underrecorded, their omission would push the apparent scaling exponent below the SST prediction, exactly as observed. The hypothesis is archaeologically plausible: multi-storey domestic architecture is independently attested in the PPNB [88], and rooftops served as a functional extension of domestic space in central Anatolian settlements such as Aşıklı Höyük and Çatalhöyük [89]; all 28 multi-storey buildings documented in the Neolithic dataset come from sites with private intramural storage and none from sites with communal storage. We tested this hypothesis by doubling the recorded floor area of buildings in Privatised sites whose total area exceeded a given threshold, repeating the simulation across thresholds from 0.5 to 2.5 ha. Across this range, the corrected scaling exponent β stabilises between 0.26 and 0.33, close to the SST prediction. Statistical significance is not reached given the small size of the Privatised sub-sample (*n* = 10), so this result is a plausibility test rather than a confirmation: it shows that the Privatised sub-sample becomes consistent with SST behaviour once vertical space is taken into account, while the Communal sub-sample remains outside the SST framework regardless of how vertical space is treated.

Hypotheses (i) and (ii) are not in competition but describe two facets of the same historical process. The privatisation of storage and the vertical elaboration of domestic space are co-occurring expressions of a single transformation: the consolidation of the household as a discrete unit of production and consumption. The shift from communal to private storage marks the moment at which a residential group takes direct control over its own surplus; the development of vertical architecture marks the moment at which the same residential group invests in expanding the productive capacity of its own dwelling.

This is why the same sub-sample of sites supports both hypotheses. The sites with privatised storage are the sites that contain every multi-storey building in the dataset; and they are also the sites for which the corrected scaling exponent approaches the SST prediction. The sites with communal storage show none of these features. The convergence is not a coincidence but a reflection of the fact that all three properties—private storage, vertical architecture, and scaling consistent with SST—are material expressions of the same underlying shift in household organisation. The implication is that SST does not fail in the Neolithic because it is empirically wrong, but because the Neolithic sample aggregates settlements at different stages of this transition. Where the household has crystallised as an autonomous productive unit, SST recovers the expected scaling once vertical space is accounted for. Where it has not, neither the proxy nor the relationship applies—not because the data are incomplete, but because the unit that SST presupposes does not yet exist.

### Time and space as independent dimensions of scaling variation

The scaling relationships documented above are not structured solely by chronology. Regional differences in urbanisation trajectories generate an independent axis of variation that cannot be reduced to period effects alone. Adding geography to the chronology-only model significantly improves fit, as does adding chronology to the geography-only model, confirming that the two dimensions structure scaling independently. This result reflects the fundamental heterogeneity of the Southwest Asian record: the same period label encompasses vastly different levels of social and economic integration across regions. During the Chalcolithic, settlements in northern Mesopotamia such as Tell Brak had already achieved urban scale and developed institutional complexity [90], while contemporary communities in Cyprus remained part of a pre-urban society where social differentiation—though present—did not consolidate into persistent inequalities [71]. The contrast persists into later periods: by the third and second millennia BCE, southern Mesopotamian cities of hundreds of hectares operated within an urbanised landscape of competing city-states connected by canal networks and long-distance trade [91,92]. In the southern Levant, the scale and character of urbanism differed markedly throughout the Bronze Age: even at its peak during the Middle Bronze Age, most settlements remained small, and during the Late Bronze Age they functioned largely as administrative centres of fragmented polities under Egyptian hegemony [93]. Indeed, how far Southwest Asia can be considered an integrated economic sphere is itself historically contingent. The ubiquity of painted pottery styles during the Halaf and Ubaid periods, the Uruk expansion of the fourth millennium BCE, the Old Assyrian trade network of the early second millennium BCE, the Late Bronze Age international system, and the Neo-Assyrian empire each demonstrate different scales and intensities of economic integration, which often coexisted with local systems operating at very different levels of connectivity within the same chronological period. This heterogeneity makes the chronological signal recovered above all the more notable, since it cannot be a mere by-product of pooling dissimilar regions within each period. Where the data permit estimation within a single region and period—eight of the twenty region × period combinations—the same temporal pattern reappears: house size bears no significant relationship to settlement size in the Neolithic (Anatolia, Levant) or the Chalcolithic (Anatolia, Cyprus), and scaling emerges in the Bronze Age, reaching significance independently in both the Levant (β = 0.725) and Northern Mesopotamia (β = 0.210) (S7 Table in S1 File). The two further estimable cells do not alter this picture: the Iron Age Levant is positive but falls just short of significance (β = 0.391), and Bronze Age Cyprus is essentially flat and too imprecisely estimated to be informative (β = 0.054, SE = 1.41). That scaling surfaces even within individual regional sequences indicates that the agglomeration signal is strong enough to be detected without the statistical power gained by combining regions.

The regional ICC values (the proportion of total variance in house size attributable to differences between site phases rather than variation within them) capture one consequence of these divergent trajectories. In Mesopotamia, ICC values are low and most variation in house size occurs within sites, consistent with the internal differentiation characteristic of economically diverse urban settlements. In Cyprus and the Levant, ICC values are higher and most variance is attributable to differences between site phases. This contrast partly reflects the composition of the sub-samples. The Levantine dataset is dominated by small Iron Age settlements under 1 ha, including sites such as Beer-Sheba, Tell en-Nasbeh, and Tell Beit Mirsim, where large domestic exposures are available thanks to the region’s tradition of biblical archaeology [38]. These small sites tend to be internally homogeneous, even though larger settlements in the sample such as Ugarit exhibit substantial internal differentiation. The Mesopotamian sub-sample includes a wider range of site sizes and periods, yielding lower ICC values that reflect greater within-site variability.

These patterns have a direct implication for the application of SST to the ancient world. Settlement scaling theory has been tested in contemporary and archaeological contexts spanning five continents, but in each archaeological application to date, settlement systems were examined within relatively coherent cultural and ecological zones (see Introduction). Our results demonstrate that in a region as diverse as Southwest Asia, aggregate scaling parameters risk conflating fundamentally different urbanisation processes unless both temporal and spatial variation are explicitly modelled. This finding also has broader implications for the GINI Project’s global comparative framework [48], within which our Southwest Asian data are situated. It suggests that regional analyses that do not account for internal heterogeneity in urbanisation trajectories may obscure rather than illuminate the relationship between settlement scale and socioeconomic outcomes.

### Mesopotamian cases: Inequality, institutions, and the city

SST proposes that scaling parameters reflect the geometry of social interaction in space and should be largely independent of specific institutional arrangements [6,8]. Our results are broadly consistent with this prediction: the scaling exponent β does not increase systematically with political complexity, and no pairwise comparison between consecutive polity groups is significant (S9 Table in S1 File). At finer resolution, however, the picture is more nuanced. When the seven individual polity types are examined separately, significant scaling emerges only for local polities (β = 0.24) and empires (β = 0.29), while intermediate categories yield non-significant exponents. This pattern is likely driven in part by limited sample sizes in the intermediate categories (7–13 site-phases for Big Man, Simple and Complex Chiefdoms, compared to 29 for Local Polities and 28 for Archaic States), and we cannot exclude the possibility that scaling would emerge with larger samples. Nevertheless, it is notable that the two polity types where scaling is detected correspond to settings where SST’s preconditions are most clearly met. Local polities represent relatively small autonomous communities where residents interact intensively within a shared settlement space, while empires encompass urban centres where economically diverse populations generate dense interaction networks. However, the intraclass correlation tells a different story entirely: ICC decreases steadily from family-level communities (0.65) through archaic states (0.41) to empires (0.22), indicating that in more complex polities a progressively larger share of the total variance in house sizes is generated within rather than between sites. This pattern is consistent with increasing functional specialisation across settlement systems. In other words, as polities grow in scale, urban centres become internally diverse—encompassing a wide range of household types from élite residences to modest houses—while rural or dependent settlements remain comparatively homogeneous and uniformly modest in residential investment. It is notable that the sharpest drop in ICC occurs between archaic states and empires, suggesting that the city-state systems of the Bronze Age, despite their urban character, may not have generated the same degree of inter-site differentiation as imperial systems, where the concentration of administrative, commercial, and productive functions at major centres drove pronounced internal variation alongside increasingly uniform peripheral settlements. In this perspective, the scaling model’s aggregate relationship with settlement size is institution-independent, while the variance structure underlying it is not.

This divergence between the aggregate scaling relationship and its consequences for residential inequality is sharpened by a direct analysis of within-site house size distributions. The Gini-size correlation is confined to high-complexity polities and, within these, to major centres at the upper tiers of settlement hierarchies. A threshold at approximately 10 ha separates sites with inequality levels comparable to family-level communities (mean Gini = 0.285) from those exhibiting pronounced residential dispersion (mean Gini = 0.551). Crucially, the scaling residuals (ξᵢ) do not predict inequality. That is, a site’s deviation from the expected scaling relationship tells us nothing about how unequally its houses are distributed. We interpret this to mean that scaling and inequality are both associated with settlement size, but through different mechanisms, with scaling generated by the geometry of interaction in space, and inequality by the institutional arrangements that govern how the returns from agglomeration are allocated among households [21,78]. In our Southwest Asian dataset, the institutional configurations that mediate the relationship between agglomeration and inequality (e.g., palace-based redistribution, temple economies, mercantile networks) represent a relatively narrow range of governance forms, predominantly towards the autocratic end of the collective-autocratic spectrum.

The emergence of scaling in the Bronze Age coincides with a fundamental transformation in the character of urbanism itself. During the Late Chalcolithic, the first large centres in Southwest Asia—Uruk in the south, Tell Brak and Khirbet al-Fakhar in the north—grew not as compact nucleated settlements but as dispersed constellations of discrete neighbourhoods, interspersed with open areas, orchards, and industrial zones [79,94,95]. Settlement hierarchies rarely exceeded two tiers, and urban growth was essentially bounded by local population concentration. Whatever the reach of Late Chalcolithic exchange networks in prestige goods and raw materials—copper, obsidian, lapis lazuli moving between polities that had developed considerable complexity through largely indigenous processes [96]—the scale a centre could sustain was ultimately determined by what its immediate agricultural catchment could provision in staple food production. In the north, the frictional cost of overland transport set a structural ceiling on centre size, such that the largest Late Chalcolithic settlements were unlikely to have exceeded the area supportable from a single site territory [30,97]. In the south, the canal network that would eventually dissolve this constraint, by reducing friction through waterborne transport, was still developing [79].

The Bronze Age broke these constraints through fundamentally different mechanisms in north and south. In the south, a demographic nucleation concentrated up to 78% of the recorded population into settlements larger than 10 ha [98], creating the dense city-state systems visible in the settlement surveys of the alluvial plain [29,99]. Low-friction waterborne transport along the Tigris-Euphrates network allowed cities to draw on agricultural surpluses from well beyond their immediate hinterlands, dissolving the size ceiling that had constrained earlier centres [79,97]. In the north, the slowly growing dispersed hubs of the fourth millennium gave way to rapidly nucleating upstarts whose growth decoupled from local population density altogether, a structural break that signals the emergence of supra-local political and economic networks, new institutional forms, and, crucially, new modes of labour organisation [27,30]. The widespread adoption of wool-bearing sheep converted vast tracts of steppe marginal to cereal agriculture into productive territory for textile production and exchange, while equids provided overland transport capacity for the resulting commodities [100], together offering a less efficient but viable alternative to waterborne trade in staples. This transition from dispersed to nucleated urbanism, and from locally bounded to regionally integrated settlement systems, created precisely the bounded, densely inhabited settlement spaces within which SST’s core mechanism—the intensification of face-to-face interaction as a function of population density—could operate. Frustratingly, data constraints prevent an analysis of scaling for sites in the very earliest urban phases during the Late Chalcolithic and Uruk periods. Here we have shown how such an analysis could be conducted when appropriate data become available. Within this newly nucleated urban landscape, the internal organisation of individual settlements reflects the institutional structures through which agglomeration effects were mediated. The earliest cases belong to the urban landscape of Early Bronze Age southern Mesopotamia, where pioneering settlement surveys documented dense constellations of city-states across the alluvial plain [29,99]. For example, Abu Salabikh (10 ha, Gini = 0.31), a secondary centre on the Euphrates channel in the Nippur region, exhibits low inequality and internally homogeneous house sizes, consistent with its position as a secondary centre in a settlement landscape characterised by extreme nucleation, where the majority of the population concentrated in cities of 40 ha or more [101,102]. Conversely Tell Asmar, the capital of the Diyala city-state (132 ha, Gini = 0.51), shows moderate inequality across its residential quarters despite being over thirteen times larger. Unlike the primate settlement hierarchy of southern Sumer, the Diyala region retained a fuller range of secondary centres alongside its dominant city [103], Tell Asmar whose dense urban fabric represents the classic form of Early Bronze Age Mesopotamian urbanism [80]. The difference between the two sites is not simply one of scale: Abu Salabikh’s houses cluster narrowly around the mean with no evidence of the pronounced size differentiation that typically accompanies economic specialisation, whereas Tell Asmar’s residential quarter exhibits a range of house sizes which might be associated with a functionally differentiated urban population. Even so, inequality at Tell Asmar remains notably lower than at comparably sized or even smaller cities in later periods, a pattern consistent with the redistributive structure of third-millennium institutional households, where intersecting lines of temple, palace, and community authority mitigated residential differentiation [25,98,104].

When this redistributive framework fragmented with the collapse of the Ur III state at the end of the third millennium BCE, new forms of economic organisation emerged. In the south, palace and temple households increasingly delegated productive activities to semi-independent operators [104], while in the north, the Old Assyrian polity developed a fully entrepreneurial trading system [105]. Kültepe-Kanesh, the Anatolian terminus of the Old Assyrian trade network (200 ha, Gini = 0.78 and 0.69 for Levels II and Ib), exhibits high inequality consistent with the socially stratified composition of its merchant quarter, where Assyrian trading families and local Anatolian households coexisted within a single urban fabric [106]. Old Babylonian Ur (100 ha, Gini ≈ 0.72), whose urban landscape extended well beyond its walled core into suburbs that accommodated lower-density residential and productive activities [107], reflects a mixed economy in which temple leases, private enterprise, and overseas trade to Dilmun operated in parallel [108]. By the first millennium BCE, high inequality is a defining feature of major urban centres. Neo-Assyrian Assur (73 ha, Gini = 0.90), which by the first millennium served primarily as the religious and ceremonial centre of the empire while political power had shifted to Kalhu and later Nineveh [109], exhibits the highest Gini in our sample. Neo-Babylonian Babylon (900 ha, Gini = 0.84), the largest walled city in the ancient world, shows comparably high inequality despite being over twelve times larger, with multiple overlapping institutional hierarchies including temple estates, state industries and private merchant houses operating in parallel [39,110,111]. The differences in Gini values across all four of these centres are small relative to sampling uncertainty and should not be over-interpreted; with sample sizes of between 27 and 60 buildings, stochastic variation alone could account for much of the observed range. What these cases collectively demonstrate—across more than a millennium of institutional transformation—is that major urban centres in politically complex systems consistently exhibit high inequality regardless of their specific economic organisation, whether structured around long-distance trade networks, mixed temple-private economies, imperial administration, or the diversified institutional landscape of a Neo-Babylonian metropolis. Whether the apparent differences between these sites reflect genuinely different distributional outcomes, or whether they are artefacts of sample size and excavation coverage, cannot be resolved with the present data.

These cities also outlast the political systems within which they are embedded. Across Southwest Asia, urban settlements persist on average over three times longer than states and empires [112]. The inequality patterns we document are thus snapshots of cities passing through successive institutional configurations, each potentially mediating the agglomeration-inequality relationship differently. At the global scale, Lawrence et al. [73] show that the association between inequality and persistence strengthens with systemic complexity and is strongest at the upper tiers of hierarchies, the same sites where our analysis identifies the strongest Gini-size correlation. Our evidence confirms that the scaling framework correctly identifies settlement size as an engine of agglomeration-driven growth, but that the distributional consequences of agglomeration are filtered through institutional structures whose specific effects cannot be fully resolved with the present sample size.

### Scaling and inequality: Implications for theory

**Fig 5:**
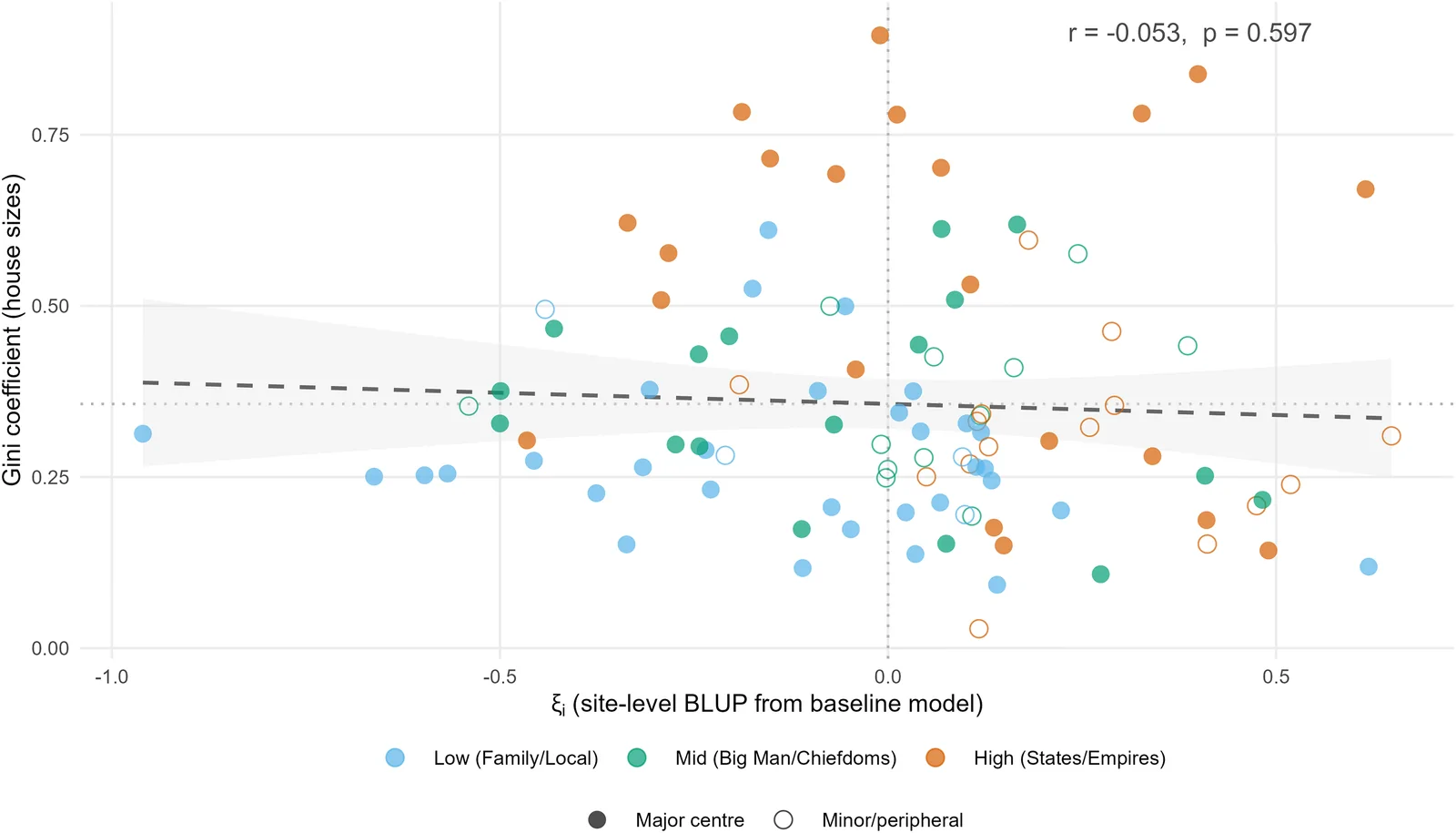
Site-level scaling residuals (ξᵢ) plotted against Gini coefficients of house sizes for site-phases with five or more buildings. Colours indicate polity group; filled circles = major centres (*HP* ≥ 0.7), open circles = minor/peripheral sites. Dashed line = linear fit with 95% CI. *r* = −0.053, *p* = 0.597.

The findings presented above have implications that extend beyond Southwest Asia. SST attributes variation in socioeconomic outcomes across settlements of similar size to the residuals ξᵢ, treating them as settlement-specific deviations from an otherwise regular scaling relationship. SST does not generate predictions about within-settlement distributions, a limitation that is already acknowledged [18]. Nevertheless, a natural question is whether settlements whose houses are larger than predicted by the scaling relationship—those with positive ξᵢ—also exhibit greater internal differentiation; if the same factors that elevate aggregate output also amplify disparities, ξᵢ should correlate with inequality. Our data allow a direct test, and the answer for Ancient Southwest Asia is negative, meaning we can say that the scaling residuals bear no relationship to inequality, either overall or within any polity group, and the null result is robust to alternative dispersion metrics (Fig 5; S10 Table in S1 File). The broad temporal spans covered by each polity group mean that this null result cannot be fully disaggregated into contemporaneous subsets with the present sample sizes, but no polity group and period combination for which testing is possible shows a positive ξᵢ-Gini relationship. In other words, the mechanisms generating scaling and the mechanisms generating inequality are disconnected. Both are associated with settlement size, but they operate through independent channels.

This result is expected, as SST does not currently address the distribution of economic activities and outcomes within cities, and addressing such questions will require refinements that move beyond mean-field predictions to incorporate contextual variables including culture, institutions, and place [18]. Our analysis provides an empirical demonstration of what this theoretical gap means in practice, in that agglomeration effects, as formalised by SST, capture the geometry of interaction in space and predict how average returns scale with settlement size, but they do not predict—and their residuals do not encode—how those returns are distributed among households. The political economy of distribution operates through a different set of variables entirely. Thompson et al. [113] reach a convergent conclusion from a different analytical perspective, showing that neighbourhood-level productivity tracks settlement-level productivity closely, while neighbourhood-level inequality is systematically lower than settlement-level inequality, a pattern consistent with the proposition that the scaling of productivity and the scaling of inequality are governed by distinct processes.

What our scaling analysis adds to the picture emerging from the GINI Project—whose database includes the Southwest Asian house size data contributed by the present authors—is a formal test of whether the aggregate scaling relationship itself contains information about distributional outcomes. The result is that it does not. The ξᵢ null result demonstrates that a settlement’s position relative to the scaling baseline tells us about its aggregate productivity, not about its internal structure. In our data, the Gini-size correlation appears only where hierarchical institutional structures concentrate the returns from agglomeration at major centres, while population scale without such institutions—as in our low- and mid-complexity polities—does not generate measurable residential inequality. This finding also challenges the common assumption in economics that productivity and inequality should scale together simply because there is more wealth available for uneven distribution. While agglomeration does increase aggregate output, the process through which that output is distributed among households intercedes, making the relationship between productivity growth and inequality far from automatic. Ortman et al. [77] situate this finding within a broader framework, identifying settlement hierarchy, population scale, and land-limited production as the principal drivers of inequality across the archaeological record, and showing that agglomeration and productivity account for only a small fraction of the global variance in Gini values. For example, the Levantine portion of our dataset, where small Iron Age settlements show uniformly low inequality despite land-limited production regimes [50], illustrates a context in which the institutional conditions for inequality amplification are absent and the scaling relationship—though present—carries no distributional consequences. The scaling framework holds across both Mesopotamia and the Levant, what differs is everything that the scaling framework does not capture.

The broader implication is that SST clearly identifies the geometric and energetic constraints governing how average socioeconomic outputs scale with settlement size and does so with remarkable consistency across contexts as different as pre-Hispanic Mesoamerica, the Roman world, and Ancient Southwest Asia, as well as a wide range of modern urban systems. Our results suggest that the aggregate scaling relationship and its distributional consequences may respond also to different variables: the institutional arrangements governing how the returns from agglomeration are organised among households represent a dimension of variation that complements rather than contradicts the scaling parameters. This reading is consistent with work across archaeology and political science on the mediating role of governance in shaping distributional outcomes [21,78,114,115]. The relationship between productivity growth and inequality is not mechanically determined: residential inequality remained low for very long periods after the Neolithic transitions despite rising productivity [81], a finding consistent with the broader conclusion from growth economics that the direction and magnitude of the growth-inequality relationship depend on institutional context rather than on productivity increases alone [116]. Ancient Southwest Asia, with its rich institutional diversity and its deep archaeological record, offers a particularly informative setting in which to explore how scaling and distribution interact, precisely because the same scaling regime encompasses cities with uniformly high inequality but markedly different institutional configurations.

### Density scaling and the demography of Ancient Southwest Asia

The density scaling analysis allows us to address two distinct but related questions: how the geometry of settlement organisation varies across periods, and how SST can contribute to the long-standing problem of population estimation in Ancient Southwest Asia.

On the first question, the scaling exponents recovered after residential correction map closely onto the predictions of SST. The Bronze Age estimate (β = 0.79) is within one standard error of the prediction for networked settlements (5/6 = 0.833), where infrastructure networks structure the built environment, while the Iron Age estimate (β = 0.69) falls within one standard error of the prediction for amorphous settlements (2/3 = 0.667). The Chalcolithic exponent (β = 0.88) sits just above the networked prediction, consistent with the ongoing transition to formal urban organisation in the fourth millennium BCE. Only the Neolithic departs substantially from SST predictions, with an exponent (β = 0.52) below the amorphous lower bound, a result that we interpret in light of the household and storage considerations discussed above. This mapping of periods onto SST regimes does not depend on the specific values adopted for the residential correction: across the full grid of plausible endpoints each period remains within the same regime, and the values used here fall near the middle of each period’s range rather than at an extreme (S12 Table; S5 Fig in S1 File). The fact that three of four periods conform to SST predictions, despite the heterogeneity of our composite samples, suggests that the geometric and energetic constraints captured by the theory operate consistently from the early urban centres of the fourth millennium onwards.

The density estimates derived from the model intercepts also engage directly with the historical debate over population coefficients in Ancient Southwest Asia. For a 10 ha Bronze Age settlement—a scale comparable to the Main Mound at Abu Salabikh—our model predicts a density of 110 persons per hectare. This figure falls well below the lower bound of Postgate’s [43] range of 248–1205 persons per hectare for the same site, and somewhat above Steinkeller’s [117] text-based estimate of 62–80 persons per hectare for Umma. Our model thus produces densities that are intermediate between architectural maxima inferred from compact residential reconstructions and the more conservative figures derivable from cuneiform administrative records. For larger Chalcolithic settlements, our model output for a 130 ha site—corresponding to Tell Brak in the LC3–4 period as recorded in our dataset—yields approximately 23,000 inhabitants at a density of 177 persons per hectare. This estimate is closely consistent with the independent reconstruction of 17,000–24,000 inhabitants proposed by Charles, Pessin and Hald [118] for Tell Brak in the same period (100–130 ha, with extended range 55–160 ha), based on an assumed density of 150 persons per hectare [97]. The convergence of two methodologically independent estimates—ours derived from scaling-based intercepts, theirs from a fixed density coefficient applied to the observed site size—provides a measure of mutual validation despite the limited Chalcolithic sample at the upper end of our size range.

The trajectory of household density at a standardised 1-hectare settlement is the most distinctive contribution of the period-by-period analysis. Density remains stable from the Neolithic (64 HH/ha) to the Chalcolithic (63 HH/ha), drops sharply in the Bronze Age (36 HH/ha), and recovers partially in the Iron Age (42 HH/ha). The drop between the Chalcolithic and the Bronze Age coincides with the diffusion of wheeled transport across Mesopotamia during the third millennium BCE [119,120], and with the substantial increase in non-residential infrastructure that characterises Bronze Age urbanism, such as palaces, temples, public spaces, and the bureaucratic and ceremonial architecture documented across southern Mesopotamian cities. The partial recovery in the Iron Age is consistent with a return to compact urban forms in many Levantine and Anatolian centres of the period, where settlement footprints contracted relative to Bronze Age maxima while institutional infrastructure remained substantial. These shifts are invisible to single-coefficient population estimates, which by construction treat density as a constant, but are made legible by a scaling approach.

### Limitations and future directions

Several limitations of this study should be acknowledged. These fall into two broad categories, uncertainties arising from the archaeological data themselves, and uncertainties arising from the historical processes that generated settlement populations and interaction networks. The first concern is principally methodological. The scaling estimates rest on two data inputs, the measured sizes of individual houses and the estimated extent of each site-phase, each of which carries its own uncertainty. The extent to which the exponent depends on which houses happened to be excavated and measured within a given site-phase can be assessed directly. The within-site bootstrap (see Methods; Table 2) leaves the baseline exponent essentially unchanged, indicating that the relationship is not an artefact of within-site sampling. Greater uncertainty surrounds the estimation of settlement extent. Although phase-specific site dimensions are used throughout, these estimates depend on the assessments of original excavators and can be contested, particularly for multi-period tells where the extent of individual occupation phases is difficult to establish independently. Unlike the sampling problem, this source of uncertainty is potentially resolvable through additional survey, excavation, chronological refinement, and improved reconstructions of settlement boundaries.

A second limitation concerns the relationship between settlement area, population, and interaction. We argued above that archaeological settlements possess an important analytical advantage compared to modern urban environments because their physical boundaries often correspond more closely to functional social boundaries. Nevertheless, this correspondence should not be assumed to be perfect. The populations recorded archaeologically are aggregates of individuals whose daily movements and social interactions may have extended beyond the settlement perimeter, and the scale of such interactions likely varied across periods, regions, and settlement systems. This is therefore not simply a measurement problem but a process problem, since even if site boundaries and populations could be reconstructed with perfect accuracy, uncertainty would remain regarding the extent to which the archaeological settlement captures the full interaction network responsible for any observed scaling relationship. In this respect, the challenge parallels a long-standing issue in contemporary urban scaling research, where scaling exponents are known to vary according to how cities and their functional hinterlands are delineated [19]. Rather than eliminating this problem entirely, archaeological data arguably reduce it by providing spatial units that are generally closer to the relevant interaction networks than those available for modern cities. If the relationship between area and population varied systematically across periods or regions, as is plausible given the different settlement morphologies documented in our dataset (see [43] for a critical discussion of density estimates in early Mesopotamian cities; see [121] for multi-centric urban forms), the transformation of the expected scaling exponent from 1/6–1/4 would not be constant, introducing unmodelled variance into comparisons of β values across sub-samples. The documented growth in baseline house size over time (the Y₀ trend in our chronological analysis) likewise implies that population density was unlikely to have remained constant across periods, an issue that future work integrating house-size distributions with independent demographic proxies and evidence for patterns of mobility and inter-settlement interaction will need to address.

A further limitation concerns the analytical categories themselves. Our analysis of political complexity relies on Johnson and Earle’s [49] typology, which classifies societies along an evolutionary continuum from Family-level groups to Empires. While this framework provides a standardised vocabulary for cross-cultural comparison, its application to archaeological contexts raises significant problems. The category of “Big Man,” for instance, was developed from ethnographic cases in Melanesia and is poorly suited to the Iron Age Levantine settlements to which it is applied in our dataset, even if it is the most appropriate of the categories available. Tell Masos and Khirbet al-Lahun—small settlements in the Negev and Transjordan respectively—are classified as Big Man polities, yet they existed within a landscape of established kingdoms and territorial states, not within the acephalous competitive systems that the Big Man concept was designed to describe. The anomalous scaling results for this category (β = 1.53, *p* = 0.044, but based on only 6 sites and 72 buildings) are almost certainly an artefact of this misclassification rather than a meaningful signal about how Big Man societies organise residential space. More broadly, the application of ethnographic typologies to archaeological contexts risks imposing categories that obscure rather than illuminate the organisational diversity of past societies. Context-sensitive classifications, grounded in regional archaeological sequences rather than cross-cultural evolutionary stages, would better capture the institutional variation that our results show to be central to scaling dynamics. We retained the Johnson and Earle typology because it is the classification system adopted by the GINI Project database from which our polity variables are drawn [48], and because replacing it with region-specific classifications would compromise the cross-cultural comparability that makes the scaling analysis possible in the first place. The trade-off between classificatory precision and cross-cultural generality is inherent to any dataset of this spatial and particularly temporal scale. We flag it here as a limitation rather than proposing a solution, since the development of archaeologically grounded alternatives that retain comparative utility is itself a major research undertaking.

The dataset itself introduces further constraints and, although it represents the largest compilation of measured house areas from Ancient Southwest Asia currently available, it remains unevenly distributed across time, space, and settlement size. The Chalcolithic period is represented primarily by small Anatolian and Cypriot villages, with southern Mesopotamia—where the earliest cities emerged—contributing only a single site. This compositional imbalance means that the absence of scaling in the Chalcolithic cannot be confidently attributed to the absence of urban processes, but it may partly reflect the absence of urban sites from the sample. The addition of more extensive domestic exposures from Late Chalcolithic centres would substantially strengthen the analysis for this critical transitional period. Tell Brak, for instance, is present in our dataset but contributes only a few buildings despite the site’s 130 ha extent, while other major centres such as Uruk and Hamoukar are absent from the Chalcolithic sample entirely. This gap reflects the state of excavation rather than of data synthesis. At Uruk, the largest Late Chalcolithic centre in southern Mesopotamia, over a century of fieldwork has concentrated on the monumental precincts of Eanna and the Anu Ziqqurat, and the few residential exposures documented outside these areas date to the Early Dynastic and later periods, not to the fourth millennium BCE [122]. At Hamoukar, Late Chalcolithic excavations uncovered administrative buildings destroyed by warfare rather than domestic quarters, and the only extensive residential exposure dates to the late third millennium BCE [123]. Filling this gap will require excavation programmes designed to expose fourth-millennium domestic architecture at a scale sufficient for distributional analysis. The same unevenness constrains the analysis of regional variation: of the twenty region × period combinations in the dataset, only eight contain enough site-phases to estimate scaling within a single region and period. This sparsity is the reason chronology and geography are modelled as separate dimensions rather than through a fully crossed region × period interaction, and it limits the extent to which the emergence of scaling can be localised to specific regional sequences rather than inferred from the pooled record.

At the upper end of the settlement hierarchy, the dataset lacks several imperial capitals—for example Nineveh and Hattusa—whose domestic architecture, where excavated, would likely extend the range of both settlement sizes and inequality values. This absence may partly account for the difference between our baseline β (0.30) and the higher imperial-period exponent reported by Squitieri and Altaweel [37] (β = 0.35–0.41), whose dataset includes a comparable number of major urban centres but fewer small sites, producing a right-skewed sample that amplifies the apparent scaling exponent. In other words, our sample, while larger in overall size, is weighted towards smaller and more peripheral settlements, potentially underestimating the agglomeration effects that SST predicts should be strongest at the apex of settlement hierarchies. Basri and Lawrence [38] note a similar limitation and suggest that the inequality levels documented in existing studies represent minima for their respective periods.

A further consideration concerns populations who left no architectural trace. In Ancient Mesopotamian cities, substantial segments of the urban population—including dependent labourers permanently attached to temple and palace estates [104], war captives, freed slaves in ambiguous legal positions, and economically marginal individuals—occupied institutional quarters or perishable structures that are archaeologically invisible [52]. Their exclusion primarily affects Gini coefficients which underestimate true inequality [51] but could also influence scaling exponents if the proportion of such archaeologically invisible residents varied systematically with settlement size, as is plausible given that larger centres concentrated proportionally more institutional dependents. More broadly, the interpretation of house size as a proxy for household productivity assumes a correspondence between architectural and economic units that may not hold uniformly across all periods in our dataset. As discussed above, the nature of the household in Neolithic Southwest Asia differed from that of later periods, and house size may not directly index household productivity in contexts where the household had not yet emerged as an autonomous economic unit [82,83,85].

The most fundamental obstacle to advancing this line of research is, as Basri and Lawrence [38] emphasise, the simple paucity of well-published excavations of domestic architecture. Notable exceptions such as Stone’s [124] analysis of Old Babylonian Nippur demonstrate the analytical potential of large residential exposures, but such cases remain rare. The tell-based archaeology of Southwest Asia, with its deeply stratified multi-period mounds, makes the exposure of large single-phase residential areas costly in time and labour, and over 150 years of excavation have overwhelmingly prioritised monumental and public architecture over domestic quarters. Expanding the dataset will require sustained investment in the excavation and publication of residential areas, complemented by geophysical prospection and remote sensing approaches that can reveal settlement layouts without full excavation. Within this broader agenda, some specific gaps in our dataset are particularly consequential, including the near-absence of domestic exposures from Late Chalcolithic southern Mesopotamia, where the earliest cities emerged; the lack of imperial capitals such as Nineveh and Hattusa, whose inclusion would extend the range of settlement sizes and inequality values; and the limited integration of archaeological house size data with the rich textual evidence on household composition and property ownership available for periods such as the Old Babylonian, Old Assyrian, and Neo-Babylonian.

A final avenue for future research concerns the infrastructural component of settlement scaling. The present study focuses on residential space because houses are the most consistently documented archaeological feature across the full temporal and geographic range of Southwest Asia, allowing a uniquely diachronic analysis. Yet SST generates explicit predictions not only for outputs and productivity but also for infrastructure, which is expected to scale sublinearly with population. Southwest Asia is particularly well suited to investigating these predictions because many forms of infrastructure, notably canal systems, road networks, transport corridors, and public spaces, are documented archaeologically and, in some periods, textually. Preliminary analyses have demonstrated that transport and communication networks can be examined using scaling approaches, revealing relationships between infrastructural development, settlement hierarchies, and regional integration [35]. Future research could extend this perspective by combining evidence from well-excavated settlements, regional surveys, remote-sensing datasets, and textual records to examine whether infrastructural investments scaled in accordance with SST predictions and how changes in transport and irrigation networks shaped the emergence of agglomeration effects.

## Conclusions

Settlement Scaling Theory offers a framework capable of bridging the study of ancient and contemporary cities within a single quantitative language. By formalising how settlement size structures socioeconomic outcomes, SST provides archaeology with tools to move beyond typological description and engage directly with the theoretical foundations of urban science. At the same time, archaeological data offer SST the temporal depth necessary to observe how scaling relationships originate, develop, and transform. This study exploits that complementarity. By assembling the largest dataset of measured house areas from Ancient Southwest Asia, we have conducted the first fully diachronic analysis of settlement scaling in the region where urbanisation first emerged.

The main result is that scaling is not uniformly present across all periods in our dataset. In the Neolithic and Chalcolithic, house size bears no systematic relationship to settlement size, and scaling becomes detectable only from the Bronze Age onwards. Whether this trajectory reflects a genuine historical threshold—the point at which social and economic integration reached sufficient intensity to generate agglomeration effects—or partly reflects the compositional limitations of our Chalcolithic sample, which largely lacks the major urban centres of Late Chalcolithic southern Mesopotamia, remains an open question. What is clear is that scaling cannot be assumed as a given for all periods and settlement types, but it must be demonstrated empirically, and its absence in earlier periods is itself a diagnostic finding, consistent with SST’s own specification that agglomeration effects require strongly mixing networks operating within bounded spatial containers. Independently of these changes in the scaling exponent, baseline house size—captured by the model prefactor *Y*₀—more than doubled from the Neolithic to the Iron Age, documenting secular growth in baseline productivity that reflects technology-driven rather than agglomeration-driven (’Smithian’) processes, and that proceeded whether or not scaling was active.

The observed scaling exponent (β ≈ 0.30) is close to the theoretical prediction for an intensive quantity regressed against settlement area (≈ 1/4), and consistent with values reported for comparable variables in Mesoamerica and the Central Andes once the transformation from population to area as independent variable is taken into account. Within the Southwest Asian record, our estimate falls between the pre-imperial and imperial-period values reported in the only previous regional study. In this sense, rather than representing an anomaly, this convergence confirms that SST’s core predictions hold across settlement traditions as different as the dispersed communities of pre-Hispanic Mesoamerica and the compact, tell-based urbanism of Southwest Asia. At the same time, our analysis reveals that temporal and spatial variation structure scaling independently, reflecting the fundamental heterogeneity of urbanisation trajectories across the region where aggregate scaling parameters risk conflating different urbanisation processes unless both dimensions are explicitly modelled.

Perhaps most consequentially, we have shown that the relationship between settlement size and residential inequality is institutionally contingent. The positive correlation between Gini and settlement size is confined to high-complexity polities and, within these, to major centres occupying the upper tiers of settlement hierarchies. In low- and mid-complexity polities, inequality bears no relationship to settlement size. The scaling residuals (ξᵢ) do not predict inequality either, suggesting the mechanisms generating scaling and the mechanisms generating inequality operate through independent channels, the former through the geometry of interaction in space, the latter through the institutional arrangements that govern how the returns from agglomeration are distributed among households. The Mesopotamian settlements in our dataset make this distinction visible at the level of individual cases, with cities exhibiting uniformly high inequality despite markedly different institutional configurations. Here lies one source of the unexplained variance documented at global scales in the relationship between agglomeration and inequality. This is consistent with work across archaeology and political science demonstrating that the distributional consequences of economic growth depend on governance as much as on productivity increases alone.

A further contribution of this study lies in the application of the scaling framework to the long-standing question of population density in Ancient Southwest Asia. By deriving household density directly from the intercepts of period-specific area scaling relationships, rather than postulating a uniform coefficient a priori, we show that residential density was not constant across periods but varied systematically with settlement size and over time. The drop in baseline density from the Chalcolithic to the Bronze Age, and its partial recovery in the Iron Age, traces a structural reorganisation of urban space in which a growing proportion of settlement area was given over to non-residential infrastructure. Our period-specific estimates fall within the ranges proposed in the existing literature on Mesopotamian demography, but improve on them in two ways: by allowing density to vary with site size, as predicted by SST and as recognised qualitatively by previous archaeological work, and by deriving the relationship from the data rather than assuming it. The convergence between our scaling-based estimates and independent reconstructions for sites such as Tell Brak suggests that this approach offers a productive route to refining population estimates across the region. A companion study, currently in preparation, builds on this framework to generate period- and site-specific population estimates across the full dataset.

Taken together, these results demonstrate that archaeology does not merely test Settlement Scaling Theory but contributes to it, and that the properties that SST treats as foundational (i.e., the existence of scaling relationships, their exponents, and their socioeconomic correlates) are themselves products of historical processes—in this case, the transition from the dispersed, locally bounded proto-urbanism of the Late Chalcolithic to the nucleated, regionally integrated city systems of the Bronze Age—that only the deep time perspective of archaeology can reveal. Understanding when, where, and under what conditions these properties emerge is not a peripheral concern but a central question for any theory that aspires to explain how cities work. Southwest Asia, as the world’s oldest laboratory of urbanisation, offers an indispensable vantage point from which to pursue that question.

## Supporting information

S1 File Supplementary methods and results. Contains additional analyses: OLS comparison for baseline scaling (S1 Table), sensitivity analysis (S2 Table), robustness of the baseline exponent to individual sites (S3 Table; S1 Fig), chronological scaling (S4 Table; S2 Fig), geographical scaling (S5-S6 Tables), robustness of scaling to regional composition (S7-S8 Tables), scaling by political complexity (S9 Table; S3 Fig), robustness of the inequality results to the choice of dispersion metric (S10 Table; S4 Fig), population density scaling and sensitivity of the residential correction (S11-S12 Tables; S5 Fig), Neolithic absence tests (S13-S14 Tables; S6 Fig), and HP threshold sensitivity (S15 Table).
(PDF)

S2 File Inclusivity in global research questionnaire. (DOCX)
