# Supplementary material for: The emergence of urban scaling in Ancient Southwest Asia
Source: PLoS One. 2026 Aug 19;21(8):e0355479. doi: 10.1371/journal.pone.0355479 (PMC13489426; doi:10.1371/journal.pone.0355479)
Supplement: Supplementary file 1 [file pone.0355479.s001.pdf]

Supporting Information

The emergence of urban scaling in Ancient Southwest Asia

Francesca Chelazzi, Dan Lawrence

S1 File. Supplementary methods and results.

Supplementary section 1: OLS comparison for baseline scaling

To assess the impact of the mixed-effects approach adopted in the main text, we compare results from ordinary least squares (OLS) regression with those from the linear mixed-effects model. OLS treats each building as an independent observation, whereas the mixed-effects model accounts for the hierarchical structure of the data by including a random intercept for site phase, recognising that buildings within the same site phase share local conditions that influence house size.

S1 Table. Comparison of OLS and mixed-effects model estimates for baseline scaling.

Metric	OLS with palaces	Mixed with palaces	OLS without palaces	Mixed without palaces
β (scaling)	0.282	0.298	0.270	0.286
SE	0.011	0.038	0.010	0.038
R^2	0.268	0.279 (marginal)	0.268	0.277 (marginal)
p value	< 0.001	< 0.001	< 0.001	< 0.001
Effective df	1850	102.6 (Satterthwaite)	1839	103.0 (Satterthwaite)
R^2 conditional	–	0.661	–	0.690
ICC	–	0.530	–	0.571
N buildings	1852	1852	1841	1841
N site-phases	–	107	–	107

The two approaches yield nearly identical β estimates (with palaces: Δβ = 0.016; without palaces: Δβ = 0.016), confirming that the scaling relationship is robust to model choice. However, OLS standard errors are approximately 3.6× smaller than mixed-effects SE, reflecting the fact that OLS does not account for within-site dependence among buildings sharing the same site phase. The effective degrees of freedom drop from 1,850 (one per building) to approximately 103 (reflecting the 107 site phases as the true number of independent units). Despite this substantial correction, the scaling relationship remains highly significant ($p < 0.001$) in all cases.

The Intraclass Correlation Coefficient (ICC = 0.530 with palaces, 0.571 without) indicates that over half of the total variance in house size is attributable to differences between site phases. This substantial clustering validates the mixed-effects approach: OLS p-values and confidence intervals are overly optimistic because they treat buildings within the same site as independent observations when they are not. The conditional R^2 (0.661 with palaces, 0.690 without) shows that site area together with site-phase identity explains approximately two-thirds of the variance in house size, compared to 28% for site area alone (marginal R^2). The gap between marginal and conditional R^2 quantifies the contribution of local, site-specific factors (e.g. construction traditions, available materials, topographic constraints, socioeconomic organisation) that affect house sizes independently of settlement size. All substantive conclusions reported in the main text hold under both approaches and regardless of palace inclusion.

Supplementary section 2: Sensitivity analysis

To assess whether our results depend on the choice of analytical unit, we compared the mixed-effects model on individual buildings (N = 1,852; 107 site phases) with OLS regressions on site-phase means (N = 107) and site-phase medians (N = 107). Site-phase aggregations do not require mixed-effects modelling as each site phase contributes a single observation.

S2 Table. Comparison of scaling estimates across analytical units.

**Method**	N	**β**	**SE**	R^2
Individual houses (mixed-effects)	1852	0.298	0.038	0.278 (marginal)
Site-phase means (OLS)	107	0.381	0.041	0.448
Site-phase medians (OLS)	107	0.263	0.039	0.302

All three approaches confirm the fundamental pattern: $\beta < 1$ in all cases ($p < 0.001$), demonstrating that houses grow more slowly than settlements regardless of analytical choice. However, the magnitude differences are informative. Site-phase means ($\beta = 0.381$) are pulled upward by large elite houses within site phases, while site-phase medians ($\beta = 0.263$) represent the typical household experience and indicate a more modest scaling relationship. The mixed-effects estimate ($\beta = 0.298$) falls between the two, consistent with a model that weights all buildings but accounts for within-site clustering. The higher R^2 values for the aggregated approaches (0.448 and 0.302) reflect the reduction in noise achieved by averaging within site phases.

This divergence between means and medians points to substantial within-site inequality in house sizes, consistent with the findings of Basri and Lawrence [37], and suggests that elite households benefit disproportionately from the scaling effects of larger settlements. The variation across methods (β range = 0.118) reflects this within-site inequality rather than methodological instability, as all three approaches converge on the same qualitative conclusion of sub-linear scaling.

Supplementary section 3: Robustness of the baseline exponent to individual sites

To verify that the baseline scaling relationship is not driven by any single site-phase, we refitted the baseline mixed-effects model 107 times, each time omitting one site-phase (a leave-one-out, LOO, procedure), and computed formal influence diagnostics (DFBETAS and Cook's distance) using the *influence.ME* package. The scaling exponent is highly stable: across all LOO refits β ranges from 0.282 to 0.308 (full-sample β = 0.298), with a standard deviation of 0.004, and the largest change produced by removing any single site-phase is Δβ = 0.015 (Iron Age Babylon). Eight site-phases exceed conventional DFBETAS or Cook's distance cut-offs (S3 Table; S1 Fig), but none alters the exponent by more than 0.015, and the relationship remains strongly sub-linear in every case. The baseline scaling exponent is therefore not an artefact of any individual site-phase.

S3 Table. Leave-one-out robustness of the baseline scaling exponent.

N site-phases	β full	β LOO min	β LOO max	β LOO SD	Max |Δβ|	DFBETAS cutoff	Cook's D cutoff	N influential
107	0.298	0.282	0.308	0.004	0.015	0.193	0.037	8

Summary of 107 leave-one-out refits of the baseline mixed-effects model, each omitting one site-phase. β full = full-sample estimate; β LOO min/max = range of the exponent across the leave-one-site-out refits; Max |Δβ| = largest absolute change from the full-sample estimate (Iron Age Babylon). Influence cut-offs: DFBETAS > 2/√n; Cook's distance > 4/n. Eight site-phases exceed at least one cut-off, none altering β by more than 0.015.

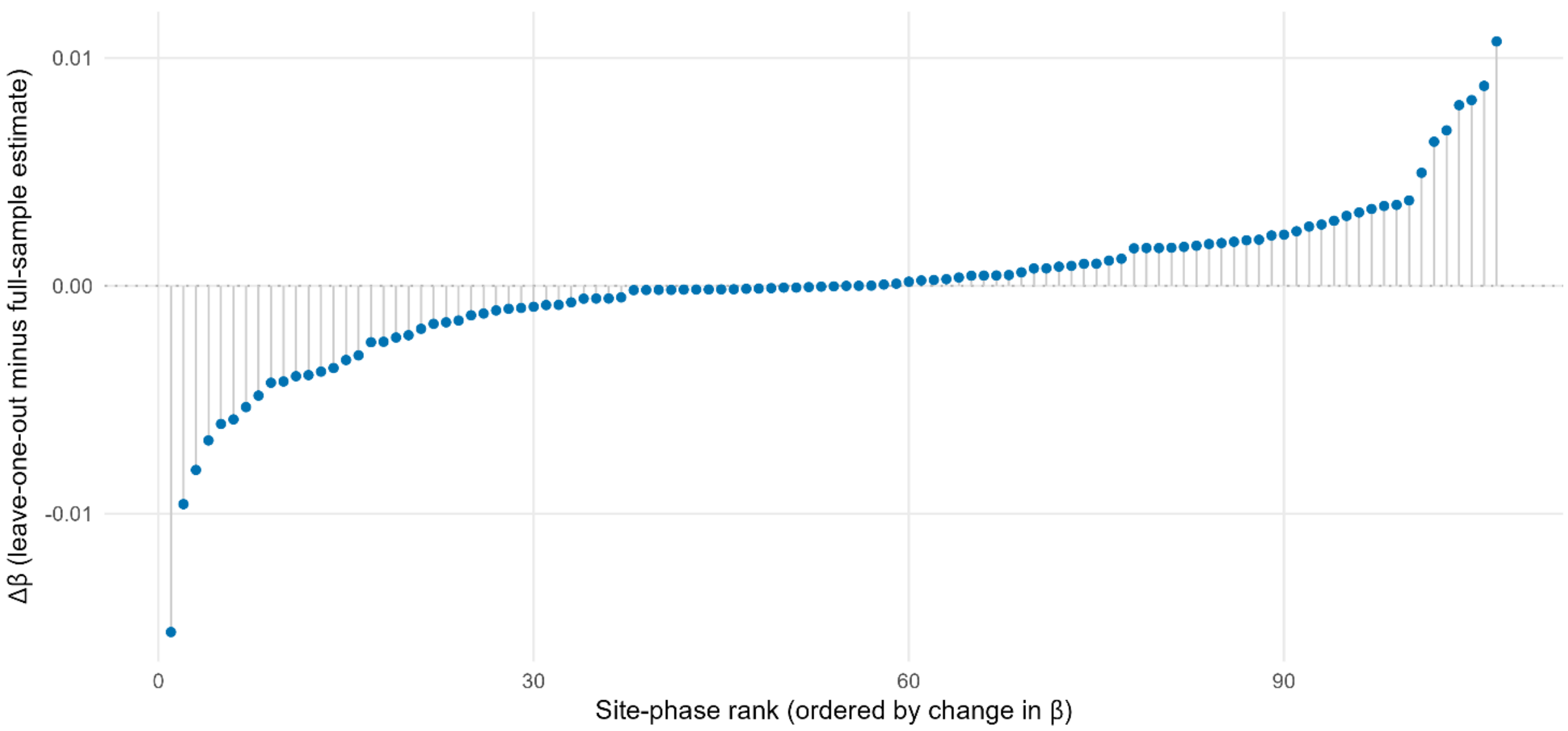

S1 Fig. Leave-one-out influence on the baseline scaling exponent. Change in β (leave-one-out estimate minus full-sample estimate) for each of the 107 site-phases, ordered by magnitude. All deviations fall within ±0.015, confirming that no single site-phase drives the baseline relationship.

Supplementary section 4: Chronological scaling analysis

Scaling exponents and prefactors were estimated separately for each chronological period using linear mixed-effects models with random intercept for site phase (Fig 2 in main text; S4 Table). The Palaeolithic (2 site phases, 14 buildings) was excluded as the number of groups is insufficient for reliable estimation of random effects. The prefactor Y_0 represents the predicted house size (m²) at a standardised settlement area of 1 ha, obtained by back-transforming the model intercept (Y_0 = 10^intercept). The secular trend in Y_0 was assessed through linear regression of the intercept against the mean midpoint date of each period (R^2 = 0.98, p = 0.012). Pairwise comparisons between consecutive periods using Wald z-tests show no individually significant transitions (all $p > 0.2$), but the cumulative increase from 16.9 to 41.8 m² is monotonic and unambiguous.

S4 Table. Chronological scaling analysis (mixed-effects model).

Period	β	SE	df	P value	R^2m	R^2c	ICC	Intercept	SE_int	Y_0 (m²)	Y_0 95% CI	N builds.	N sites	N site-phases	β boot. mean	β boot. SE	β boot. 95% CI
Neolithic	0.051	0.144	22.0	0.727	0.004	0.742	0.741	1.228	0.076	16.9	[12.0, 23.8]	330	19	24	0.051	0.046	[-0.039, 0.140]
Chalcolithic	0.097	0.120	20.6	0.427	0.016	0.511	0.503	1.359	0.087	23.0	[15.5, 34.0]	248	16	21	0.099	0.039	[0.028, 0.179]
Bronze Age	0.245	0.056	35.2	<0.001	0.199	0.471	0.340	1.502	0.071	31.8	[23.1, 43.8]	808	26	38	0.246	0.016	[0.215, 0.276]
Iron Age	0.242	0.054	20.3	<0.001	0.294	0.545	0.357	1.621	0.066	41.8	[31.0, 56.4]	452	18	22	0.244	0.020	[0.207, 0.283]

Linear mixed-effects model with random intercept for site phase, estimated separately for each period. The Palaeolithic (2 site phases, 14 buildings) was excluded as the number of groups is insufficient for reliable estimation of random effects. R^2 marginal = variance explained by site area; R^2 conditional = variance explained by site area and site-phase identity; ICC = proportion of variance attributable to between-site-phase differences. Intercept = model intercept

on $\log_{10}$ scale; Y_0 = back-transformed prefactor (10^intercept), representing predicted house size in m² at a settlement area of 1 ha; 95% CI computed as 10^(intercept ± 1.96 × SE_int). β bootstrap columns report the mean, standard error and 95% percentile interval of the scaling exponent from a within-site bootstrap (1,000 replicates, houses resampled within each site phase).

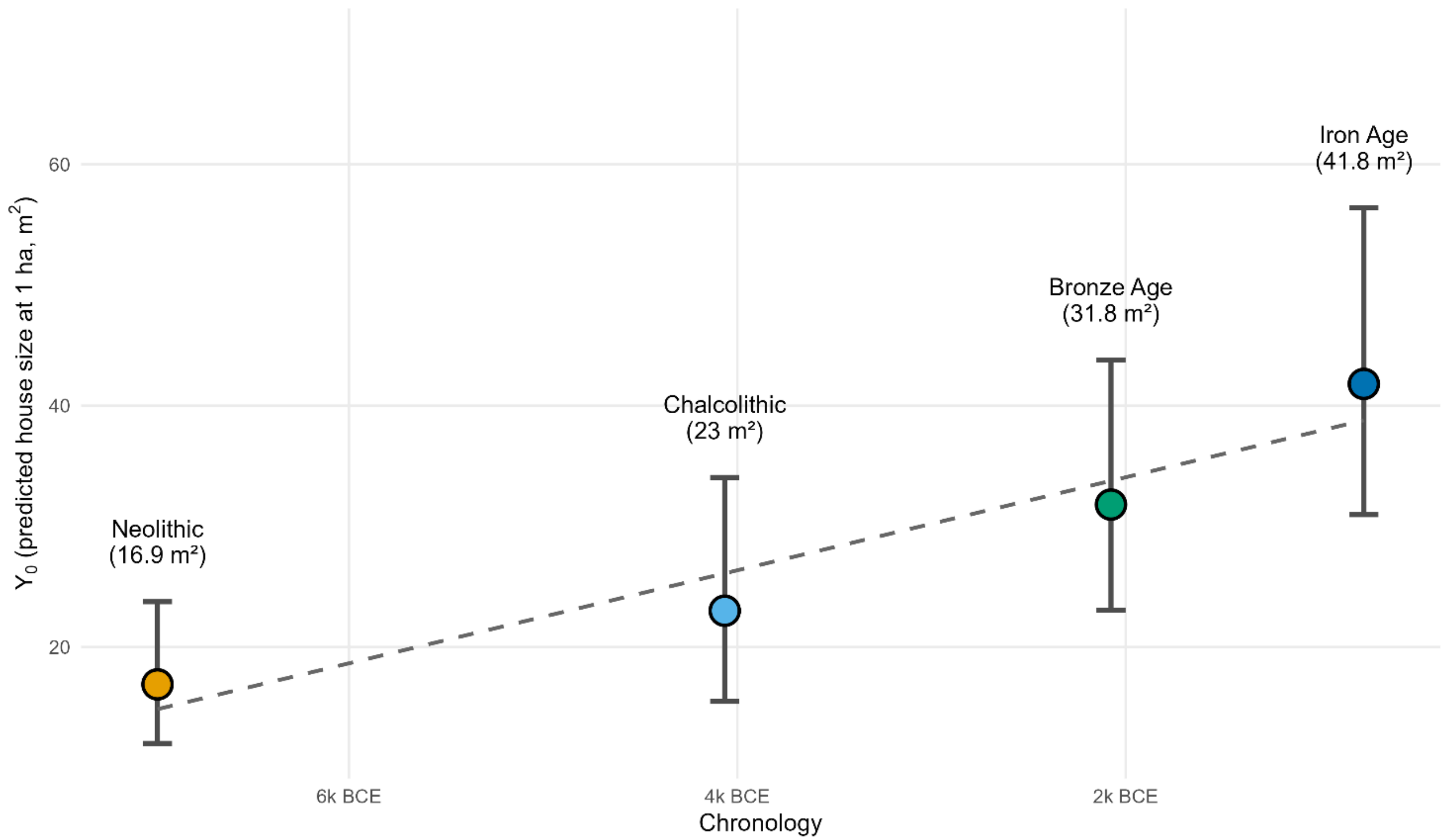

S2 Fig. Prefactor Y_0 (predicted house size in m² at a standardised settlement area of 1 ha) plotted against the mean midpoint date of each period. Error bars represent 95% CI, computed as 10^(intercept ± 1.96 × SE). The monotonic increase from 16.9 m² (Neolithic) to 41.8 m² (Iron Age) is highly linear (R^2 = 0.98, p = 0.012).

Supplementary section 5: Geographical scaling analysis

Scaling exponents were estimated separately for each geographical region using linear mixed-effects models with random intercept for site phase (S5 Table). Each region's sample has a markedly different chronological composition, as shown in the building count table below (S6 Table), which partly accounts for the variation in scaling exponents across regions.

S5 Table. Geographic scaling analysis.

Region	β	SE	df	*p* value	R^2 marg.	R^2 cond.	ICC	*N* builds.	*N* sites	*N* site-phases
Anatolia	0.297	0.062	17.4	< 0.001	0.380	0.593	0.344	434	16	23
Cyprus	0.248	0.177	12.6	0.186	0.115	0.750	0.717	222	10	15
Levant	0.443	0.107	28.8	< 0.001	0.252	0.736	0.647	661	26	31
N. Mesopotamia	0.180	0.050	25.3	0.001	0.132	0.350	0.251	384	20	28
S. Mesopotamia	0.199	0.081	9.9	0.034	0.135	0.335	0.231	151	9	10

Linear mixed-effects model with random intercept for site-phase, estimated separately for each region. R^2 marginal = variance explained by site area; R^2 conditional = variance explained by site area and site-phase identity; ICC = proportion of variance attributable to between-site-phase differences. The chronological composition of each regional sample differs substantially (see below), which partly accounts for the variation in scaling exponents.

S6 Table. Number of buildings by region and chronological period.

Region	Palaeolithic	Neolithic	Chalcolithic	Bronze Age	Iron Age
Anatolia	—	146	111	110	67
Cyprus	—	85	65	72	—
Levant	14	57	—	268	322
N. Mesopotamia	—	34	57	260	33
S. Mesopotamia	—	8	15	98	30

Supplementary section 6: Robustness of scaling to regional composition

To assess whether the overall scaling relationship is driven by the regional composition of the sample, and whether the chronological trajectory holds within regions, we carried out two analyses. First, we estimated the scaling exponent separately for each region × period cell with at least five site-phases, reporting cells below this threshold as insufficient data (S7 Table). Of the twenty region × period combinations, only eight met the threshold, reflecting the uneven distribution of domestic exposures across space and time that motivates our treatment of chronology and geography as separate dimensions. Where estimation was possible, the results reproduce the overall pattern: scaling is absent in the Neolithic (Anatolia $\beta = -0.089$, Levant $\beta = -0.062$, both n.s.) and Chalcolithic (Anatolia $\beta = 0.299$, Cyprus $\beta = 0.073$, both n.s.), and emerges in the Bronze Age, where it is significant in both the Levant ($\beta = 0.725$, $p = 0.005$) and Northern Mesopotamia ($\beta = 0.210$, $p = 0.011$), remaining positive into the Iron Age in the Levant ($\beta = 0.391$, $p = 0.069$). The emergence of scaling is therefore detectable within individual regions and not solely a product of pooling across them.

Second, we refitted the baseline model omitting one region at a time (leave-one-region-out, LOO; S8 Table). The exponent is stable, ranging from 0.272 to 0.335 against a full-sample value of 0.298. The largest change follows removal of Northern Mesopotamia ($\beta = 0.335$, $\Delta\beta = 0.037$), whose comparatively low regional exponent and large Bronze Age sample pull the pooled estimate downward; no other region shifts β by more than 0.026. The baseline scaling relationship is thus not an artefact of the over- or under-representation of any single region.

S7 Table. Chronological scaling within regions.

Region	Period	N site-phases	N builds.	β	SE	p value	Note
Anatolia	Neolithic	9	146	−0.089	0.309	0.781	
	Chalcolithic	10	111	0.299	0.273	0.305	
	Bronze Age	2	110	—	—	—	insufficient data
	Iron Age	2	67	—	—	—	insufficient data
Cyprus	Neolithic	4	85	—	—	—	insufficient data
	Chalcolithic	5	65	0.073	0.171	0.699	
	Bronze Age	6	72	0.054	1.41	0.970	singular fit; unstable
Levant	Neolithic	6	57	−0.062	0.394	0.883	
	Bronze Age	8	268	0.725	0.163	0.005	
	Iron Age	15	322	0.391	0.197	0.069	
N. Mesopotamia	Neolithic	4	34	—	—	—	insufficient data
	Chalcolithic	4	57	—	—	—	insufficient data
	Bronze Age	18	260	0.210	0.074	0.011	
	Iron Age	2	33	—	—	—	insufficient data
S. Mesopotamia	Neolithic	1	8	—	—	—	insufficient data
	Chalcolithic	2	15	—	—	—	insufficient data
	Bronze Age	4	98	—	—	—	insufficient data
	Iron Age	3	30	—	—	—	insufficient data

Mixed-effects models with a random intercept for site phase, estimated separately for each region × period cell with ≥ 5 site-phases; cells below threshold reported as insufficient data. The Cyprus Bronze Age estimate derives from a singular model and should be treated as unstable. The Palaeolithic is omitted, so the site-phase counts in this table do not sum to the regional totals used in the LOO analysis (S8 Table).

S8 Table. Leave-one-region-out robustness of the baseline scaling exponent.

Region removed	N site-phases removed	β LOO	Δβ
Northern Mesopotamia	28	0.335	+0.037

Southern Mesopotamia	10	0.272	−0.026
Anatolia	23	0.294	−0.004
Cyprus	15	0.295	−0.003
Levant	31	0.297	−0.001
None (full sample)	0	0.298	0

Baseline mixed-effects model refitted with each region (Subarea) removed in turn. β LOO = exponent on the reduced sample after removing one region (leave-one-region-out); Δβ = change from the full-sample estimate (β = 0.298).

Supplementary section 7: Scaling by political complexity

Scaling exponents were estimated separately for each polity type following Johnson and Earle's [48] classification, using linear mixed-effects models with random intercept for site phase (S3 Fig; S9 Table).

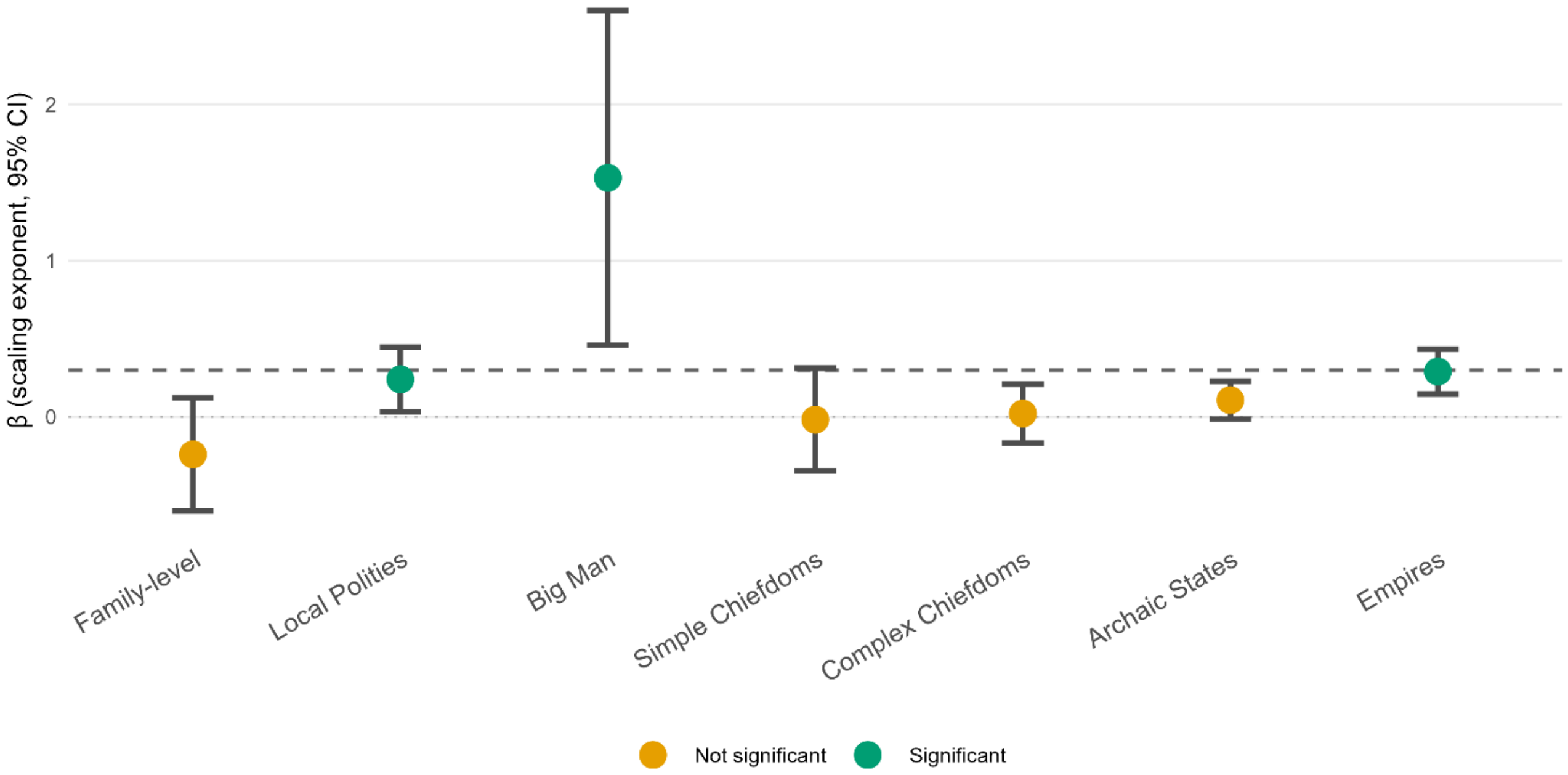

S3 Fig. Scaling exponents (β) with 95% confidence intervals for each polity type. The dashed line indicates the overall baseline β (0.298); the dotted line marks β = 0. The Big Man category is an artefact of a small, heterogeneous sample (6 sites, 72 buildings) and should not be interpreted as a meaningful scaling relationship.

Only Local Polities (β = 0.240) and Empires (β = 0.289) exhibit significant and interpretable scaling, while the nominally significant Big Man result (β = 1.53) is an artefact of a small, heterogeneous sample. The remaining types show no significant relationship between house size and settlement size. Pairwise comparisons between consecutive types reveal no significant differences, indicating that political complexity does not systematically alter scaling exponents. For analytical clarity, polity types were amalgamated into three groups (S9 Table).

S9 Table. Scaling by polity group (mixed-effects model).

Polity Group	β	SE	df	*p* value	R^2 marg.	R^2 cond.	ICC	*N* builds.	*N* sites	*N* site-phases
Low	0.230	0.102	36.8	0.030*	0.081	0.693	0.666	533	29	39
Mid	0.160	0.099	30.1	0.116	0.040	0.516	0.496	464	24	31
High	0.178	0.048	33.9	< 0.001	0.142	0.453	0.362	855	29	37

Low = Family-level and Local Polities; Mid = Big Man, Simple and Complex Chiefdoms; High = Archaic States and Empires.

The Low and High groups show significant scaling, while the Mid group does not, although the Low group's significance is borderline (p = 0.030) and should be interpreted with caution given the small effective sample size (39 site phases). Scaling exponents do not increase systematically with political complexity: Low is slightly higher than High, and pairwise differences between consecutive groups are not significant. The ICC, however, decreases progressively from Low to High, indicating that more complex polities exhibit greater within-site variation in house sizes. This pattern suggests that while political complexity does not

alter the average rate of house size scaling, it does increase internal socioeconomic differentiation, a finding explored through the Gini analysis in the main text.

Supplementary section 8: Robustness of the inequality results to the choice of dispersion metric

As robustness checks on the Gini-based inequality analysis, we computed two alternative dispersion metrics within each site phase (*N* = 101 site phases with at least five measured buildings): the variance of $\log_{10}$(house area), which is more directly connected to the log-normal distributional assumptions of SST than the Gini coefficient, and the P90/P10 ratio of house areas, a measure based on the tails of the size distribution.

Overall, the variance of $\log_{10}$(house area) correlates positively with settlement size ($r = 0.325$, $p < 0.001$), confirming that larger sites exhibit greater dispersion in house sizes. The pattern across polity groups replicates the Gini results precisely: the correlation is significant only in high-complexity polities ($r = 0.504$, $p = 0.002$), with no significant relationship in low-complexity or mid-complexity polities. The P90/P10 ratio follows the same direction—positive only in high-complexity polities (r = 0.271)—but does not reach significance (p = 0.110), reflecting the greater sampling noise of an extreme-percentile ratio computed on relatively few buildings per site phase.

S10 Table. Correlation of two dispersion metrics with $\log_{10}$(site area), by polity group.

**Polity Group**	***r* (log-variance)**	***p* value**	***r* (P90/P10)**	***p value***	***n***
Low (Family/Local)	−0.091	0.598	0.047	0.787	36
Mid (Big Man/Chiefdoms)	−0.131	0.498	-0.117	0.544	29
High (States/Empires)	0.504	0.002	0.271	0.110	36

Correlations for the P90/P10 ratio are computed on $\log_{10}$(ratio). Only site phases with at least five measured buildings are included.

The three dispersion metrics are strongly convergent (Gini-log-variance $r = 0.887$; Gini-P90/P10 $r = 0.800$; both $p < 0.001$), indicating that they capture essentially the same underlying construct. The consistency of the polity-group pattern across all metrics confirms that the institutional contingency of the size-inequality relationship documented in the main text is not an artefact of the choice of inequality measure.

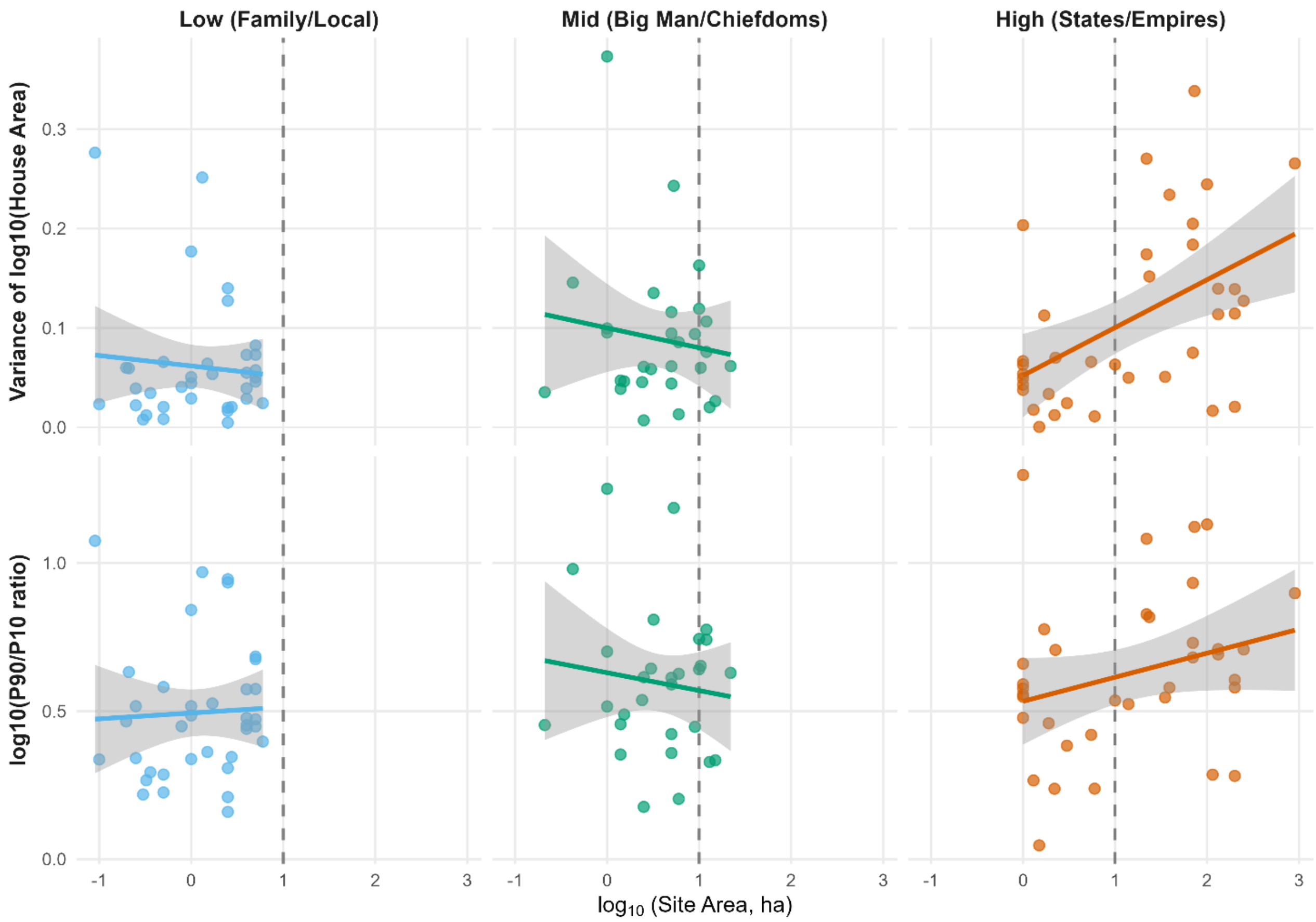

S4 Fig. Two dispersion metrics plotted against settlement area ($\log_{10}$), faceted by polity group: variance of $\log_{10}$(house area) (top row) and $\log_{10}$(P90/P10 ratio) (bottom row). In both metrics the positive relationship is confined to high-complexity polities, replicating the Gini pattern shown in Fig 3 of the main text.

Supplementary section 9: Population density scaling and sensitivity of the residential correction

The density scaling analysis presented in the main text reports residential-corrected exponents and population estimates for each chronological period. The full set of estimates, including both uncorrected and residential-corrected versions, is reported here for transparency (S11 Table). Uncorrected estimates derive from fitting $\log_{10}(TotalHH) = \alpha + \beta \times \log_{10}(TotalArea)$ directly to the window-extrapolated household counts. Residential-corrected estimates apply a residential-proportion correction that scales log-linearly from 0.90 at small sites (1 ha) to 0.60 at large sites (100 ha) for the Chalcolithic, Bronze Age, and Iron Age, following Stone [43]; the Neolithic is left uncorrected, as the dispersed character of large Neolithic settlements is already reflected in the window-based estimate [61]. The residential correction reduces the scaling exponent in the three later periods, bringing the Bronze Age and Iron Age estimates within one standard error of the SST prediction for networked (5/6) and amorphous (2/3) settlements respectively, while the Chalcolithic remains slightly above the networked prediction. The Neolithic is unaffected, since no correction is applied. Back-transformed intercepts (HH per hectare at a settlement area of 1 ha) decrease accordingly under the corrected version, reflecting the smaller residential proportion assumed for larger sites.

S11 Table. Density scaling exponents and intercepts by period, with and without residential-proportion correction.

Period	*N* site-phases	β uncorr	SE	*p* value	HH/ha uncorr	β corr	SE	*p* value	HH/ha corr
Neolithic	24	0.517	0.171	0.006	64.4	0.517	0.171	0.006	64.4
Chalcolithic	18	0.950	0.197	< 0.001	71.9	0.880	0.196	< 0.001	63.4
Bronze Age	38	0.866	0.114	< 0.001	40.3	0.789	0.113	< 0.001	35.7
Iron Age	16	0.775	0.198	0.002	46.4	0.694	0.198	0.003	41.8

HH/ha = back-transformed intercept (predicted households per hectare at a settlement area of 1 ha). For the Neolithic, no residential correction is applied; uncorrected and corrected values therefore coincide. Ordinary least squares fitted at the site-phase level. Kerkenes Dağ excluded.

Because the residential correction rests on two assumed endpoints—the residential proportion at small sites (1 ha) and at large sites (100 ha)—we tested how sensitive the corrected exponent is to these choices. We varied both endpoints independently across a grid from 0.50 to 1.00, retaining only combinations in which the residential proportion does not increase with site size, and refitted the per-period density model for every combination (S12 Table; S5 Fig). The corrected exponent is highly stable: across the entire grid it ranges from 0.834 to 0.950 in the Chalcolithic, 0.733 to 0.866 in the Bronze Age, and 0.646 to 0.775 in the Iron Age—a total spread of roughly 0.12–0.13 per period, smaller than the gap between the two SST regimes themselves (networked 5/6 = 0.833 versus amorphous 2/3 = 0.667). In every case the period remains within the same SST regime: the networked prediction falls within the Bronze Age range and the amorphous prediction within the Iron Age range, while the Chalcolithic stays at or above the networked prediction throughout. Moreover, the values adopted in the main text (0.880, 0.789 and 0.694 respectively) lie near the middle of each period's range rather than at an extreme (S5 Fig). The SST classification of each period is therefore not an artefact of the specific residential-correction values adopted in the main text.

S12 Table. Sensitivity of the corrected density exponent to the residential-correction assumptions.

Period	β min	β max	β baseline	SST prediction
Chalcolithic	0.834	0.950	0.880	0.833 (networked)
Bronze Age	0.733	0.866	0.789	0.833 (networked)
Iron Age	0.646	0.775	0.694	0.667 (amorphous)

Corrected exponent obtained by varying the residential proportion at small sites (1 ha) and large sites (100 ha) independently over a grid from 0.50 to 1.00 (combinations with non-increasing proportion only). β baseline = value under the correction adopted in the main text (0.90 at 1 ha, 0.60 at 100 ha). The Neolithic is excluded (no correction applied). SST predictions are discussed in the main text; the Iron Age sample pools Levantine city-states and Mesopotamian imperial centres, so its amorphous classification reflects an average across settlement systems.

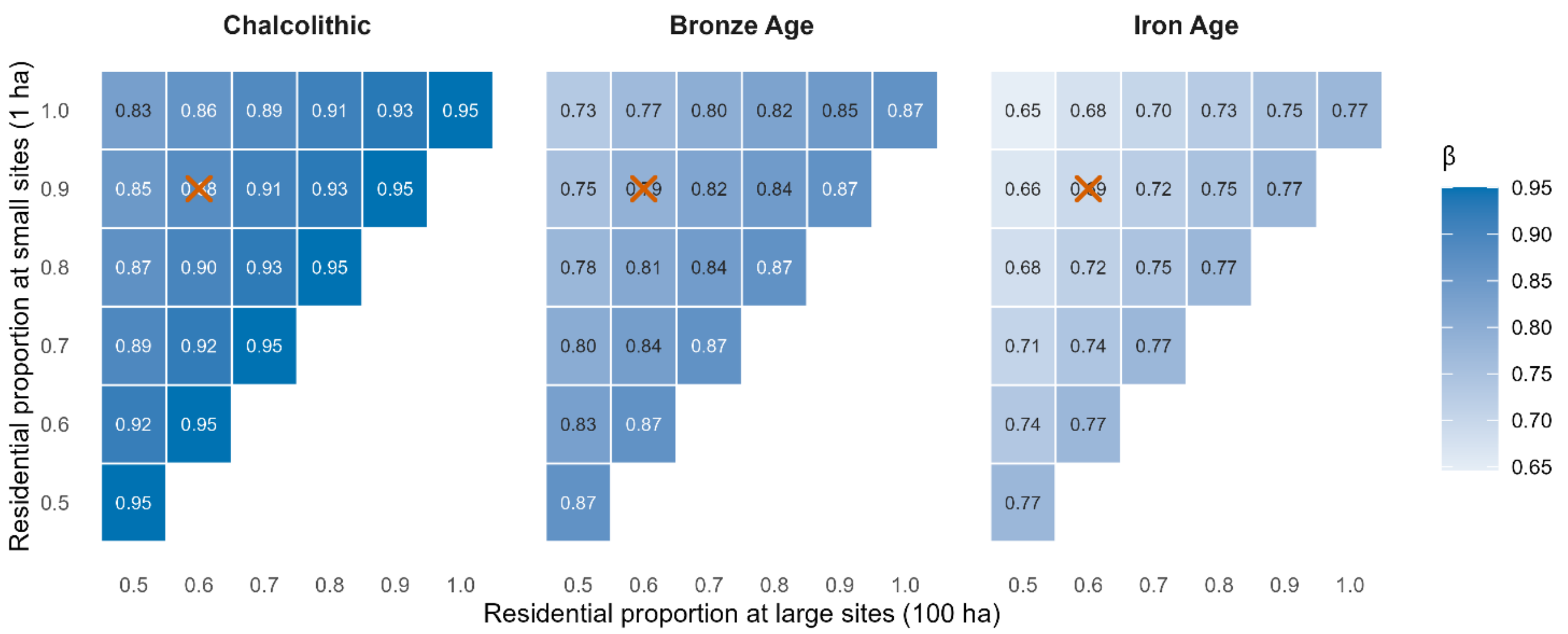

S5 Fig. Sensitivity of the corrected density exponent to the residential-correction assumptions. Corrected scaling exponent (β) for each period across the grid of residential-proportion endpoints; the x-axis is the proportion assumed at large sites (100 ha) and the y-axis at small sites (1 ha). Only combinations in which the residential proportion does not increase with site size are shown. The orange cross marks the correction adopted in the main text (0.90 at 1 ha, 0.60 at 100 ha). The Neolithic is omitted, as no correction is applied to it.

Supplementary section 10: Tests on the Neolithic absence of scaling

The absence of significant scaling in the Neolithic period (see Fig 2 and S4 Table) was investigated through three diagnostic tests, each addressing a substantive hypothesis discussed in the main text: (i) that the available data systematically underrepresent vertical architecture; (ii) that house size in the Neolithic does not function as a proxy for household productivity in the same way as in later periods; and (iii) that the Neolithic sample aggregates regions with different subsistence baselines. The results of these three tests are reported below in the order in which the corresponding outputs appear.

Test 1: Centering by sub-groups (Hypothesis iii)

To test whether the absence of Neolithic scaling reflects the aggregation of regional sub-systems with different productivity baselines, we re-fitted the area scaling relationship ($\log_{10}$ *TotalHH* against $\log_{10}$ *TotalArea*) after centering the data by Subregion, Subarea, and Subarea × Period (S6 Fig). For each centering scheme, both predictor and response were transformed by subtracting the within-group mean, removing baseline differences across sub-systems while preserving the slope of the relationship within each group. The Neolithic exponent remains non-significant under all centering schemes, while Bronze Age and Iron Age scaling remain robust regardless of the scheme applied. Hypothesis (iii) is therefore excluded.

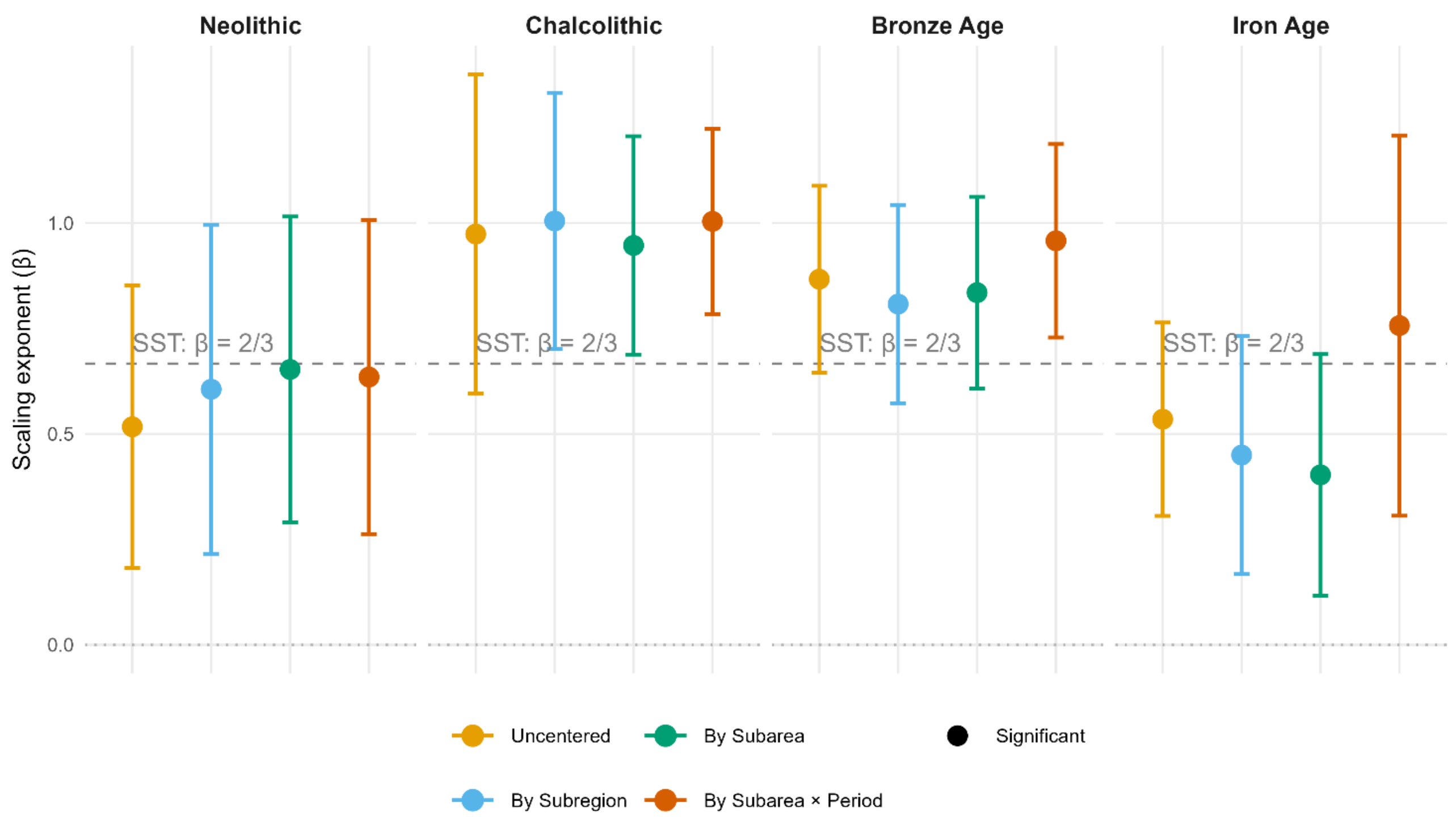

S6 Fig. Scaling exponents (β) with 95% confidence intervals for each chronological period under different centering schemes (Uncentered, By Subregion, By Subarea, By Subarea × Period). The dashed line marks the SST prediction (β = 2/3) for area scaling at the aggregate level; the dotted line marks β = 0. Filled circles denote statistically significant relationships ($p < 0.05$); open circles denote non-significant relationships.

Test 2: Storage regime classification (Hypothesis ii)

To test whether house size in the Neolithic functions differently as a proxy for household productivity, we classified all 22 Neolithic site-phases (17 sites) in our dataset according to their household storage regime, on the basis of published archaeological evidence for the presence or absence of dedicated intramural storage features. Site-phases where storage was predominantly communal (extramural, shared granaries, with no dedicated storage spaces within houses) were classified as Communal (n = 12); sites with dedicated

intramural storage and controlled access were classified as Privatised (n = 10). Scaling was then re-fitted within each group using mixed-effects models with random intercept for site phase (S13 Table). Neither group exhibits significant scaling, but the intercept (Y_0) more than doubles between Communal and Privatised site-phases, indicating that house size in the Neolithic tracks the storage regime rather than settlement scale.

S13 Table. Scaling within Neolithic storage regimes.

Storage regime	*N* site-phases	*N* buildings	β	SE	*p* value	Y_0 (m²)
Communal	12	191	0.092	0.145	0.540	12.0
Privatised	10	124	-0.013	0.252	0.960	30.8

Y_0 is the back-transformed intercept (predicted house size at a settlement area of 1 ha). Mixed-effects models with random intercept for site phase.

Test 3: Simulation of unrecorded vertical space (Hypothesis i)

To test whether the absence of Neolithic scaling reflects systematic underrepresentation of vertical architecture, we simulated unrecorded multi-storey construction by doubling the recorded house area for buildings in Privatised sites above varying site-area thresholds (0.5 to 2.5 ha; S14 Table). The simulation reflects the assumption that vertical expansion of domestic architecture was both archaeologically more frequent and more often unrecorded in larger Neolithic settlements with private intramural storage, where storage privatisation is associated with the emergence of two-storey building plans (e.g., Beidha, Bouqras, Çayönü Cell Plan). The result is robust across the range of thresholds tested: β stabilises in the range of 0.26 to 0.33, close to the SST prediction. Although none of the simulated values reaches statistical significance, given the small sample size of the Privatised sub-sample (n = 10), this exercise demonstrates that a modest correction in the right places—applied only to larger sites with private storage—is sufficient to recover the expected scaling pattern. This finding aligns with the observation that all 28 multi-storey buildings documented in the Neolithic dataset come from sites with private intramural storage, none from sites with communal storage, supporting the interpretation that vertical expansion was a structural correlate of storage privatisation.

S14 Table. Simulation of unrecorded vertical architecture in Privatised Neolithic sites.

Threshold (ha)	Site-phases doubled	Site-phases total	β	SE	*p* value	Y_0 (m²)
None (baseline)	0	10	-0.013	0.252	0.960	30.8
0.5	8	10	0.262	0.233	0.295	50.8
1.0	7	10	0.302	0.275	0.306	47.2
1.5	5	10	0.334	0.277	0.263	41.0
2.0	5	10	0.334	0.277	0.263	41.0
2.5	5	10	0.334	0.277	0.263	41.0

House area is doubled for buildings in sites above each site-area threshold. The thresholds 1.5-2.5 ha produce identical partitions of the sample because no Privatised sites have area in this range. Mixed-effects models with random intercept for site phase.

Taken together, these three tests indicate that the absence of significant scaling in the Neolithic is unlikely to be an artefact of regional heterogeneity (Hypothesis iii excluded by Test 1), and is consistent with two complementary processes: the underrepresentation of vertical architecture in the available record (Hypothesis i, partially supported by Test 3) and the differential applicability of house size as a proxy for household productivity in pre-Neolithic-PPNB economies (Hypothesis ii, supported by Test 2).

Supplementary section 11: Sensitivity analysis for the Hierarchy Position (HP) threshold

To identify the *HP* threshold that best discriminates between sites where inequality scales with settlement size and sites where it does not, we conducted a sensitivity analysis varying the cutoff between 0.5 and 0.8 within high-complexity polities (S15 Table).

S15 Table. Sensitivity analysis for the HP threshold within high-complexity polities (Archaic States and Empires).

Threshold	*N* major	*N* minor	Mean Gini major	Mean Gini minor	*r* major	*p* major	*r* minor	*p* minor
0.5	31	5	0.438	0.405	0.628	< 0.001	-0.156	0.802
0.6	29	7	0.444	0.389	0.626	< 0.001	-0.330	0.469
0.7	21	15	0.526	0.303	0.439	0.047	-0.087	0.758
0.8	18	18	0.521	0.345	0.272	0.275	0.685	0.002

For each threshold, sites with HP ≥ threshold are classified as "major centres" and the remainder as "minor/peripheral". *r* = Pearson correlation between Gini coefficient of house sizes and $\log_{10}$(site area).

The positive correlation between inequality and settlement size among major centres is significant across thresholds from 0.5 to 0.7 (*r* = 0.44-0.63, *p* ≤ 0.047), though it weakens as the threshold rises and the major-centre group shrinks, while minor/peripheral sites show no significant relationship at any of these thresholds. At 0.8, the correlation among major centres is no longer significant (r = 0.27), while a spurious positive correlation appears among minor sites (r = 0.69), because functionally important centres with *HP* between 0.7 and 0.8 are reclassified as peripheral. The threshold of 0.7 is therefore adopted throughout the main text as the empirical upper bound at which the distinction between primary centres and dependent settlements holds.
