# Supplementary material for: The emergence of urban scaling in Ancient Southwest Asia
Source: PLoS One. 2026 Aug 19;21(8):e0355479. doi: 10.1371/journal.pone.0355479 (PMC13489426; doi:10.1371/journal.pone.0355479)
Supplement: Supplementary file 2 [file pone.0355479.s002.docx]

Inclusivity in global research

PLOS’ policy on inclusivity in global research aims to improve transparency in the reporting of research performed outside of researchers’ own country or community and ensures that PLOS publications reporting global research adhere to high standards for research ethics and authorship. Authors of relevant research articles may be asked to complete the questionnaire below, which outlines ethical, cultural, and scientific considerations specific to inclusivity in global research. This questionnaire may be requested when researchers have travelled to a different country to conduct research, if research uses samples collected in another country, research with Indigenous populations or their lands, or if research is on cultural artefacts. Researchers travelling to another country solely to use laboratory equipment will not normally be required to complete the questionnaire. However, the questionnaire can be requested at the journal’s discretion for any submission – if you have been requested to complete this questionnaire by the PLOS journal you submitted to, please do so.

Please complete the questionnaire below and include this as a Supporting Information file with your manuscript. Note that if your paper is accepted for publication, this checklist will be published with your article in the supporting information files. Please ensure that you reference the checklist in the main body of your manuscript. We suggest adding a subsection ‘Inclusivity in global research’ to your Methods section and adding the following sentence: “Additional information regarding the ethical, cultural, and scientific considerations specific to inclusivity in global research is included in the Supporting Information (SX Checklist)”

The questions have been designed to be applicable to a wide range of study types, and there are subsections for both human subjects research and non-human subjects research. If any of the questions are not relevant to your research please mark them as “N/A” as appropriate.

**Ethical considerations, permits and authorship**

*This section is applicable to all research types.*

Provide details as to who granted permissions and/or consent for the study to take place in the Methods section of your manuscript. This should include the names of **all** ethics boards, governmental organizations, community leaders or other bodies that provided approval for the study. If individuals provided approval refer to these people by their role or title but do not list their name(s).

Reported on page number: N/A — no permits required (secondary analysis of previously published data). See Methods, p. 12, lines 269–272 (tracked-changes version).

If there were any deviations from the study protocol after approval was obtained please provide details of these changes in the Methods section of your manuscript.
Did this study involve local collaborators that are residents of the country where the research was conducted or members of the community studied? If you do not have any authors from said communities, please provide an explanation for this below.

Reported on page number: NA

This study is a secondary analysis of previously published archaeological data and did not involve new field research in the countries of origin; it therefore did not require local data collection or local collaborators for its execution. The underlying measurements derive from decades of excavation and publication carried out under the relevant national authorities and by researchers working in and on the region, whose primary studies are cited throughout the manuscript. The data were compiled within the international GINI Project research network. The two authors are based at institutions in the United Kingdom and Italy and meet PLOS' authorship criteria on the basis of their contributions to the conception, analysis, and writing of this study.

Everyone listed as an author should meet PLOS’ criteria for authorship and all individuals who meet these criteria should be included in the author byline, rather than the acknowledgements. For further information please see the journal’s Authorship Policy.

**Human subjects research (e.g. health research, medical research, cross-cultural psychology)**

Did you obtain written informed consent from a representative of the local community or region before the research took place? How did you establish who speaks for the community? Details of written informed consent obtained from study participants should be reported separately in the Methods section of your manuscript.

N/A — this study did not involve human subjects, participants, or direct engagement with any community. It is a secondary analysis of previously published archaeological data (measurements of residential architecture).

How did members of the local community provide input on the aims of the research investigation, its methodology, and its anticipated outcome(s)?

N/A (no human subjects research; secondary analysis of previously published archaeological data).

When engaging with the local community, how did you ensure that the informed consent documents and other materials could be understood by local stakeholders?

N/A (no human subjects research; secondary analysis of previously published archaeological data).

Will the findings of the research be made available in an understandable format to stakeholders in the community where the study was conducted (e.g. via a presentation, summary report, copies of publications, etc.)? Please provide details of how this will be achieved.

The article will be published open access under a CC-BY licence in PLOS ONE, and the full dataset, analysis code, and executable notebook are openly deposited in Zenodo (doi:10.5281/zenodo.20172243), freely accessible to all, including researchers and institutions in the region. No community-specific dissemination applies, as the study is a secondary analysis of previously published data and did not involve human subjects.

**Non-human subjects research using specimens/ animals collected as part of the study, or those housed in archival collections. Examples include archaeology, paleontology, botany and zoology.**

Did the permission you obtained from a local authority to perform the study include an agreement on access to outputs and benefit sharing? This may include procedures to enable fair distribution of the benefits and resources arising from the research performed. Please include any details of Prior Informed Consent and Benefit Sharing Agreements obtained. These may be required by field-specific regulations, for example the Convention on Biological Diversity (CBD) and the associated Nagoya Protocol.

N/A — No permission from a local authority was obtained or required, as no specimens or biological materials were collected: this study is a secondary analysis of previously published measurements of residential architecture. Prior Informed Consent and Benefit Sharing Agreements (e.g. under the CBD/Nagoya Protocol) are therefore not applicable.

If the material used in your study was imported, please A) provide the year it was imported and B) indicate whether permits were obtained to import/export the materials used, C) provide details of any permits obtained. If this information is not available, please indicate this.

N/A. No physical materials were imported or exported. This study uses previously published measurements of residential architecture compiled from site reports and monographs; no specimens or materials were physically handled, transferred, or acquired by the authors.

If you used archival specimens, please state how the material used in your study was acquired by the institute it is held in and provide details of any permits obtained for the original excavations/ sample collection. If this information is not available, please indicate this.

N/A. No archival specimens or physical materials were examined. The study uses previously published measurements of residential architecture (building floor areas) drawn from site reports, monographs, and the GINI Project database; no objects, samples, or curated collections were accessed by the authors.

How was the potential cultural significance of the materials collected in your study to local communities considered in your research design? Were Indigenous peoples and/or local researchers and institutions involved with archaeological excavations / collection of specimens? If so, please provide a description of their involvement.

This study did not involve any new excavation or collection of materials by the authors; it is a secondary analysis of previously published measurements of residential architecture. The primary archaeological data were generated by earlier fieldwork carried out under the relevant national authorities of each country and by researchers and institutions working in and on the region, whose studies are cited throughout the manuscript. The cultural significance of the built heritage of Southwest Asia is recognised: the analysis operates at the level of aggregate, already-published architectural measurements (building floor areas), and does not involve culturally sensitive materials, human remains, or objects of ritual or symbolic value. Because the study is a re-analysis of existing published data rather than new field research, the involvement of Indigenous peoples and local researchers pertains to the original excavations documented in the cited primary sources, not to the present study.

If your manuscript includes photographs of human remains please indicate whether authors obtained permission from descendants or affiliated cultural communities to do so.

N/A — The manuscript does not include photographs of human remains, nor any human remains. It presents analyses of residential architecture (building floor areas) only.
